# Doula-Delivered Cognitive Behavioral Training and Cardiovascular Health Intervention for Birthing Individuals in a Low-Income New York City Population: Protocol for a Living Healthy for Moms Randomized Type I Hybrid Effectiveness-Implementation Trial

**DOI:** 10.2196/76871

**Published:** 2025-12-10

**Authors:** Jennifer M Faiz, Imogen Bylinsky, Lorena Rincones Rojas, Katherine Kopatsis, Victoria St. Clair, Madeleine Dorval-Moller, Kristin Voegtline, Heather Lipkind, Monika M Safford, Kelli Stidham Hall, Uma Reddy, Lauren M Osborne

**Affiliations:** 1 Department of Obstetrics and Gynecology Weill Cornell Medicine New York, NY United States; 2 The Bridge Directory Brooklyn, NY United States; 3 Northern Manhattan Perinatal Partnership (NMPP) New York, NY United States; 4 Department of Obstetrics and Gynecology, Department of Population Health Sciences Weill Cornell Medicine New York, NY United States; 5 Department of Medicine Weill Cornell Medicine New York, NY United States; 6 Departments of Social, Behavioral, & Population Sciences; Epidemiology Celia Scott Weatherhead School of Public Health & Tropical Medicine Tulane University New Orleans, LA United States; 7 Department of Obstetrics and Gynecology Columbia University Irving Medical Center New York, NY United States; 8 Department of Obstetrics and Gynecology, Department of Psychiatry Weill Cornell Medicine New York, NY United States

**Keywords:** postpartum, mental health, cardiovascular disease, doula, cognitive behavioral therapy

## Abstract

**Background:**

In the United States, mental health complications and cardiovascular events are the 2 leading causes of death for birthing parents in the year following delivery. Most of these deaths are preventable, with Black and Latinx individuals experiencing higher rates of these postpartum complications. Current postpartum care has not reduced these disparities.

**Objective:**

This randomized controlled trial aims to train nonmedical professionals in a novel intervention to prevent postpartum depressive symptoms and improve cardiovascular health following childbirth in a low-income New York City (NYC) birthing population.

**Methods:**

We aim to recruit 600 birthing individuals over 3 sites across NYC. After screening and consent, participants will be randomized to the Living Healthy for Moms intervention (doula-delivered cognitive behavioral therapy and cardiovascular behavioral health intervention) or attention control (a variation of standard postpartum doula care). Daily telephone contacts for the first 7 days immediately after hospital discharge are followed by 12 doula-led video sessions conducted over 6 months. The primary outcomes are postpartum depressive symptoms and cardiovascular health, with secondary outcomes of psychosocial status, health behaviors, health care use, and patient satisfaction. All outcomes are measured via REDCap (Research Electronic Data Capture; Vanderbilt University) surveys administered at baseline, 2 weeks, 6 weeks, 3 months, and 6 months post discharge. Physiological measurements of glycated hemoglobin (hemoglobin A_1c_), lipids, and blood pressure will be collected at baseline, 3 months, and 6 months. Doulas, hospital staff, and birthing individuals will be recruited to evaluate the implementation of the intervention following the conclusion of recruitment at each site. A mixed methods triangulated approach will be used, including electronic health record data extraction, web-based surveys, key informant interviews, and focus groups.

**Results:**

Recruitment and data collection began at the first site in Brooklyn on January 23, 2025. Data collection for participants from each of the 3 recruitment sites will be concluded by November 30, 2027, February 29, 2028, and August 31, 2029, respectively, for Brooklyn, Queens, and upper Manhattan. Thus, all data for the total expected 600 participants will be collected by the end of the grant year 6, August 31, 2029. Trial results will not be analyzed until grant year 7, beginning September 1, 2029. Data will be analyzed on an intention-to-treat basis by study team members blinded to participant conditions.

**Conclusions:**

This hybrid type 1 effectiveness-implementation randomized controlled trial will test a novel nonspecialist intervention to prevent mental health and cardiovascular health complications of childbirth, culturally adapted to our local population in NYC. We expect that our findings will contribute to knowledge on the effectiveness and implementation of nonspecialist postpartum interventions in low-resourced settings and the expansion of doula care in the post partum.

**Trial Registration:**

ClinicalTrials.gov NCT06666400; https://clinicaltrials.gov/study/NCT06666400

**International Registered Report Identifier (IRRID):**

DERR1-10.2196/76871

## Introduction

### Maternal Mortality

In the United States, mental health (MH) and cardiovascular events are the 2 leading causes of death for birthing parents in the year following delivery. It is estimated that over 84% of these maternal deaths are preventable, stressing the importance of enforcing both physical and mental hygiene during the postpartum period [1]. Over one-fourth of eclampsia cases and pregnancy-related strokes happen within the first 7-10 days post partum, and approximately 5% of individuals develop hypertension only after hospital discharge [2,3]. Overall, 40% of maternal mortality occurs within the first 6 weeks post partum, making this a high-risk period [2,3].

Some populations are at higher risk of postpartum complications. In New York state, MH conditions are 1 of the top 3 causes of postpartum deaths for Black and Latinx individuals [4]. It has been estimated that over 75% of these deaths are preventable [4]. Similarly, Black birthing individuals are at the highest risk of cardiovascular deaths in the postpartum period [5,6]. Black and Latinx individuals bear a higher cumulative burden of stress and stressful life events, which can lead to a greater likelihood of both MH and cardiovascular events developing during the perinatal period [7]. This can also lead to a greater risk of complications in subsequent pregnancies [8,9].

### Lifelong Impacts

Postpartum depression (PPD) is highly detrimental to health and overall well-being. Individuals with PPD can remain depressed for up to 11 years after delivery [10]. PPD is associated with significant medical and functional impairment, higher medical costs, obesity, a sedentary lifestyle, and smoking [11]. PPD is also linked to decreased adherence to medical and self-care procedures, as well as overall poor health behaviors [11]. For individuals with depression, there is a higher risk of death due to suicide and substance misuse [1]. PPD is also adversely related to decreased maternal care, reduced responsiveness, bonding issues, and breastfeeding challenges. These stressors can impact infants’ emotional, cognitive, and social development [12].

Individuals who experience adverse outcomes during pregnancy or delivery are at a greater risk of developing similar health conditions during future pregnancies and are at a greater risk of developing life-threatening cardiovascular conditions. Women who experience a hypertensive disorder during pregnancy have a fourfold higher risk of being diagnosed with hypertension within 6-12 months post partum [13,14]. Similarly, gestational diabetes mellitus (GDM) is the most common complication of pregnancy and confers risk for later development of type 2 diabetes mellitus (T2DM); racial and ethnic minorities are at both greater risk of developing GDM and T2DM if they have GDM. T2DM is a powerful risk factor for cardiovascular disease (CVD) [15]. These poor health outcomes can arise as early as hospitalization for delivery and can persist for more than 5 years post partum. The risk for these poor health complications is more significant for vulnerable communities, who bear a greater cumulative stress burden and have a higher lifetime risk of CVD [4].

Therefore, pregnancy is seen as a “stress” test for future CVD [8,16,17]. The steps needed to reduce the likelihood of developing CVD are reflected in the American Heart Association’s (AHA) metric of cardiovascular health, Life’s Essential 8 (LE8). LE8 has 2 categories, behavioral and physiological, with 4 components in each category. The 4 behavioral components include diet, physical activity, cigarette smoking, and sleep. The 4 physiological components include blood pressure (BP), cholesterol, blood glucose, diabetes, and body mass index [18,19]. Ideal levels on the LE8 score (defined as 80-100 on a 100-point scale) are associated with lower risk of CVD (including heart failure), cancer, mortality, adverse pregnancy outcomes, cognitive decline, and dementia, as well as high health care costs [18]. For vulnerable communities, achieving ideal levels of LE8 is extremely beneficial. For Black individuals, achieving an ideal score on one of the 8 components is associated with lower risk of cardiovascular complications (in 1 study, 40.4% vs 25.3%), and reaching 2 ideal LE8 component scores is associated with even lower risk (16.8%) [19]. To date, LE8 has not been tested in the postpartum context.

### Shortfalls in Existing Postpartum Care

Postpartum care is often underused and tends to inadequately address the mental and physical health needs of individuals post partum. Despite aggressive screening and treatment recommendations from the American College of Obstetricians and Gynecologists (ACOG) [20], limited time for visits, access barriers, and lack of provider knowledge in addressing both mental and cardiovascular health mean that fewer than one-third of people living with PPD are formally diagnosed. Of those, only 16% receive any treatment, with only 6% getting adequate care and 3% achieving remission [21]. These numbers are stark and highlight a growing need for comprehensive PPD screening and treatment. Similarly, a systematic review revealed that among birthing parents with GDM, rates of postpartum follow-up were lowest for minorities, those with limited education, and comorbid MH conditions [22]. Implementing screening for PPD can increase attendance and the effectiveness of comprehensive care visits, including monitoring for hypertension and T2DM [23]. However, marginalized communities are often not screened for PPD and have less access to comprehensive care and medical providers. Up to 25% of those in marginalized communities cannot identify their postpartum provider, and over 50% of marginalized individuals do not go to postpartum obstetric visits [23,24]. These statistics reveal a prominent inequity within current medical treatment for postpartum individuals.

Structural racism, social racism, and unconscious bias both in and out of the health care system leave many vulnerable communities unable to receive adequate and necessary postpartum care [25]. In a 2022 Community Health Needs Assessment conducted by NewYork-Presbyterian (NYP) Hospital (the health system covering 2 of the 3 sites in our trial), postpartum individuals and community organizations identified 3 shortcomings in the health care system for vulnerable populations [25]. The first was hesitancy to seek care due to past negative interactions with health care providers and the health care system. The second was the lack of accessible, affordable, convenient, and high-quality health care services, which are severely lacking in vulnerable communities. The third was a need for more doulas and community health workers to bridge the gap between the current health care system and vulnerable communities in the postpartum period; this includes (but is not limited to) more community health workers in education, screening, preventive services, and secondary and tertiary care.

The ACOG recommends that postpartum care must be treated as a continuous process tailored to each individual’s physical and MH needs [26-28]. All women should have contact with their obstetric care provider within 3 weeks post partum, with a comprehensive postpartum visit within 12 weeks post partum [29]. The postpartum visit should be considered a full assessment, covering mood and emotional well-being, infant care and feeding, sexual health, sleep and fatigue check-ups, physical recovery, chronic disease screening and management, and general health maintenance [29]. During these postpartum visits, clinical and social resources must be available, and providers should refer patients for long-term care, especially for those with conditions such as hypertension, diabetes, thyroid disorders, and mood disorders [29]. ACOG recommends follow-up within 7-10 days for women with hypertensive disorders of pregnancy, and 72 hours for those with severe hypertension [30]. However, few health care providers have implemented these recommendations. There is still no clear roadmap for managing MH and cardiovascular conditions post partum [29]. Members of these vulnerable populations often experience complex emotional and physical stressors, some of which are time-sensitive [31,32]—for example, postpartum preeclampsia, deep vein thromboses, and postpartum endometritis commonly occur during the first 7-10 days of post partum [2,3].

New York City (NYC) has increased community-based doula care to address the shortcomings of postpartum care for vulnerable communities. Doulas provide affordable nonmedical care during pregnancy, birth, and post partum. Doulas can extend physical, emotional, and informational support to people and their families [33]. They effectively increase satisfaction and lower the risk of severe maternal morbidity [34]. While doula care shows promise as a valuable resource for individuals in the postpartum period, most doula care in NYC is focused on intrapartum care, and evidence to support postpartum doula care is still evolving [35]. We propose to address these gaps by implementing and evaluating a doula-delivered intervention, Living Healthy for Moms (LHMoms), in 3 geographically distinct settings and populations (Brooklyn, Queens, and Northern Manhattan). We set out to culturally adapt an existing cognitive behavioral therapy (CBT) intervention delivered by nonspecialists for our NYC population, integrating cardiovascular health. We expected that doulas would be willing and able to receive training to deliver this intervention. The overall trial hypothesizes that participants completing the intervention will have lower prevalence of postpartum depressive symptoms (PPDS), better cardiovascular health, and greater engagement with postpartum health care than those receiving a variant of usual doula postpartum care [34,36].

## Methods

### Study Overview

LHMoms is a hybrid type 1 effectiveness-implementation trial developed to build new knowledge on alternative models of postpartum care that may better meet the needs of high-risk birthing parents. LHMoms will use a phase 1 randomized controlled trial (RCT) to test a novel, evidence-based, doula-delivered intervention that integrates CBT and LE8, compared to a variant of standard postpartum doula care. LHMoms partners with doulas from 2 community organizations: the Caribbean Women’s Health Association (CWHA) and the Northern Manhattan Perinatal Partnership (NMPP). LHMoms is based on the Thinking Healthy Programme (THP), a widely used intervention endorsed by the World Health Organization that uses nonspecialists to deliver CBT-based care in underresourced settings around the globe. THP has shown high efficacy for both treatment and prevention of PPD in numerous settings [37-39] and has also been adapted for populations enduring physical health disorders such as diabetes and knee osteoarthritis [40-42]. We began our work to adapt THP with a series of focus groups with doulas, health care providers, and birthing individuals from the communities served by our hospital sites. This study’s team (which includes community partners and doulas) then created and refined an intervention designed to address both physical health and behavioral conditions that arise post partum. The initial draft was created over several months using feedback from the focus groups.

**Figure 1:**
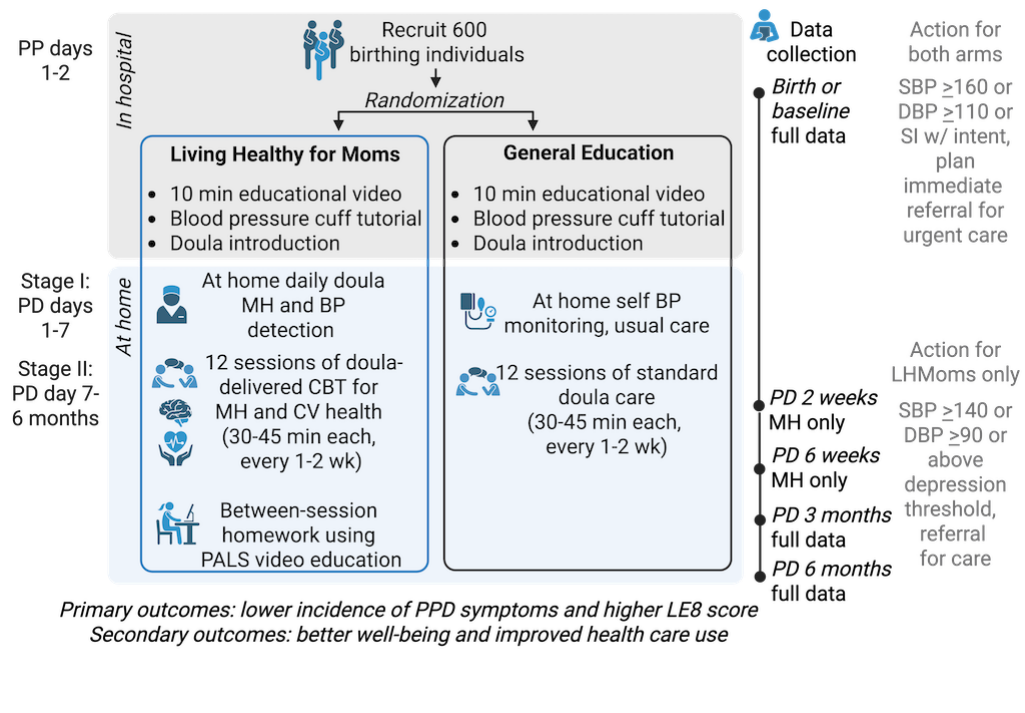
The LHMoms intervention study design includes recruitment of 600 birthing individuals and 6 months of follow-up for each participant. BP: blood pressure; CBT: cognitive behavioral therapy; CV: cardiovascular; DBP: diastolic blood pressure; LE8: Life’s Essential 8; LHMoms: Living Healthy for Moms; MH: mental health; PALS: Patient-Activated Learning System; PD: post discharge; PP: postpartum; PPD: postpartum depression; SBP: systolic blood pressure; SI: suicidal ideation; w/: with.

Participants in LHMoms are randomized to the intervention arm, where participants receive the doula-delivered CBT+cardiovascular health intervention, or the attention control arm, where participants receive a variation of standard doula-delivered postpartum care (based on a community-informed and provider-informed standardized checklist for doula care routinely used by one of our community partners, CWHA). Individuals are enrolled during delivery hospitalization and are consented and connected to their doulas in the hospital, predischarge. The intervention begins within the first week post hospital discharge and continues up to 6 months post hospital discharge. Throughout the 6 months, the intervention arm receives a total of 12 doula-led sessions. The attention control arm also receives a total of 12 doula-led sessions. Sessions for all participants are delivered via Zoom (Zoom Communications, Inc) or phone, based on the participants’ preference. All participants are fitted for and receive a home BP monitoring device and are trained on the correct way to use it before discharge. The LHMoms intervention arm begins with a 7-day emergency detection period, during which doulas contact participants daily within the first week at home to monitor MH and cardiovascular health, as the highest risk of severe maternal morbidity exists during this time period. The LHMoms study design is outlined below in Figure 1. The primary outcomes will be prevention of PPDS and cardiovascular health, measured at 6 months post partum. Doulas are supervised by this study’s psychiatrist or psychologist throughout this study, and all study data is entered into a REDCap (Research Electronic Data Capture) database maintained at the primary study site.

### Implementation Evaluation

In addition to the effectiveness trial, LHMoms aims to evaluate the success of the implementation and explore how the findings can be used to strengthen future interventions. LHMoms will use a landscape analysis, which will collect information on the implementation process and evaluate the outcomes and factors (eg, community, people, organizations, etc) that affect implementation. Doulas, hospital staff (including clinical providers and hospital administrators), and participants who have participated in the RCT will be recruited to collect data on the effectiveness of the implementation of LHMoms. To collect data, we will use a mixed methods triangulated approach, which includes electronic health record (EHR) data extraction, web-based surveys, key informant interviews, and focus groups. These data will be gathered from representative participants, including doulas, health care providers, and birthing individuals, allowing us to incorporate feedback from participants overall and specific to each site to help us further refine the intervention. Data collection and analysis will be guided by the Consolidated Framework for Implementation Research (CFIR) and RE-AIM, a framework based on 5 key components: Reach, Effectiveness, Adoption, Implementation, and Maintenance.

### Study Population, Setting, and Recruitment

This study aims to enroll 3 types of participants for the RCT and implementation process analysis. The first is doulas, who will be trained to deliver either the LHMoms intervention or the attention control variation of standard doula care to RCT participants. The second is birthing individuals participating in the RCT. The third comprises hospital staff, including clinical providers and hospital administrators, for the implementation process analysis phase. Three hospitals in NYC that serve low-income communities will be included: the NYP Brooklyn Methodist Hospital in Brooklyn, New York; the NYP Allen Hospital in upper Manhattan, New York; and the NYC Health + Hospitals Queens Hospital in Queens, New York. These sites were chosen because they serve underresourced individuals, including high numbers of Black, Latinx, low-income, and immigrant individuals.

The selection of doulas occurred before finalization of the intervention manual, approximately 8 months before RCT participant recruitment began at our first hospital site. Doulas will be selected and trained at the other 2 sites, approximately 8 months before participant recruitment, to allow time for training and certification. There will be 8 doulas per site, with 4 being assigned to the intervention arm and 4 being assigned to the control arm, for a total of 24 doulas across all sites. Purposive sampling is used in selecting doulas, who will be recommended by our community groups based on their interests and skills.

The recruitment period for the RCT will be staggered across years 2 to 6 of this study.

There will be an 18-month recruitment period at each hospital site, and we aim to recruit 200 participants per site for a total of 600 participants. At each hospital location, a community-based recruiter will be on-site. Research staff prescreen the EHR for eligible subjects and then discuss potential recruits with onsite principal investigators and hospital staff. The care team first approaches potentially eligible patients, inquires about their interest in LHMoms*,* and informs research staff about their willingness to be contacted. Research staff then approach eligible individuals within 48 hours after their delivery and before their hospital discharge.

A purposive sample of doulas, hospital staff, including clinical providers and hospital administrators, and participants will be recruited to assess the efficacy of LHMoms implementation, guided by the CFIR and RE-AIM frameworks. Ten doulas or members of health care staff at each site will either complete a web-based survey or participate in a focus group, and 7 of these will be recruited to participate in key informant interviews. Seven participants from each site will also be recruited to complete either web-based surveys or key informant interviews.

### Doula Training and Creation of Intervention

Intervention development started with this study’s team (which includes doulas and staff from our community partners) meeting with doulas to obtain advice about cultural adaptations and realistic expectations. The team then prepared a first draft of the intervention manual, based on the THP, cardiovascular health interventions previously undertaken by this study’s team, and the recommended cultural adaptations and realistic expectations. An important component of making the intervention realistic is time constraints and the realities of living with an infant, leaving little time for intervention delivery. To accommodate the need for education, the intervention relies on the theory-driven, evidence-based Patient-Activated Learning System web-based education platform, designed by team members to provide information about medical conditions for people with low health literacy and racially marginalized individuals. The education component is designed to be self-driven, with specific assignments demanding no more than 15 minutes for completion before intervention sessions. Education components deliver knowledge on education on postpartum health, cognitive behavioral strategies, cardiovascular health, and overall health and are designed to support the intervention sessions, which are more behaviorally oriented [43].

In semiweekly training sessions (1.5 hours each) across 4 months, the initial cohort of LHMoms doulas was trained in CBT and motivational interviewing (MI) principles and role-played delivery of each intervention session. Doulas also received an overview of the Patient-Activated Learning System. Simultaneously, doulas provided feedback for this study’s team, who refined and adapted the intervention iteratively, following an approach previously developed by our team [44]. Sessions were recorded, and any doula who missed a session was required to watch the recording. Following refinement of each session, doulas were paired to continue practicing the intervention with each other and with members of this study’s team. For doulas at our subsequent hospital sites, or any doulas recruited as replacements, training will begin with watching recordings of the initial training sessions, followed by a reduced number of sessions with this study’s team emphasizing CBT and MI principles, role play, and practice.

Doulas assigned to the attention control arm were trained over a total of 6 sessions, focusing on session-specific content and role-playing with no education on CBT or MI principles. They reviewed typical postpartum care topics, avoiding any discussion of topics specific to the LHMoms intervention.

**Figure 2:**
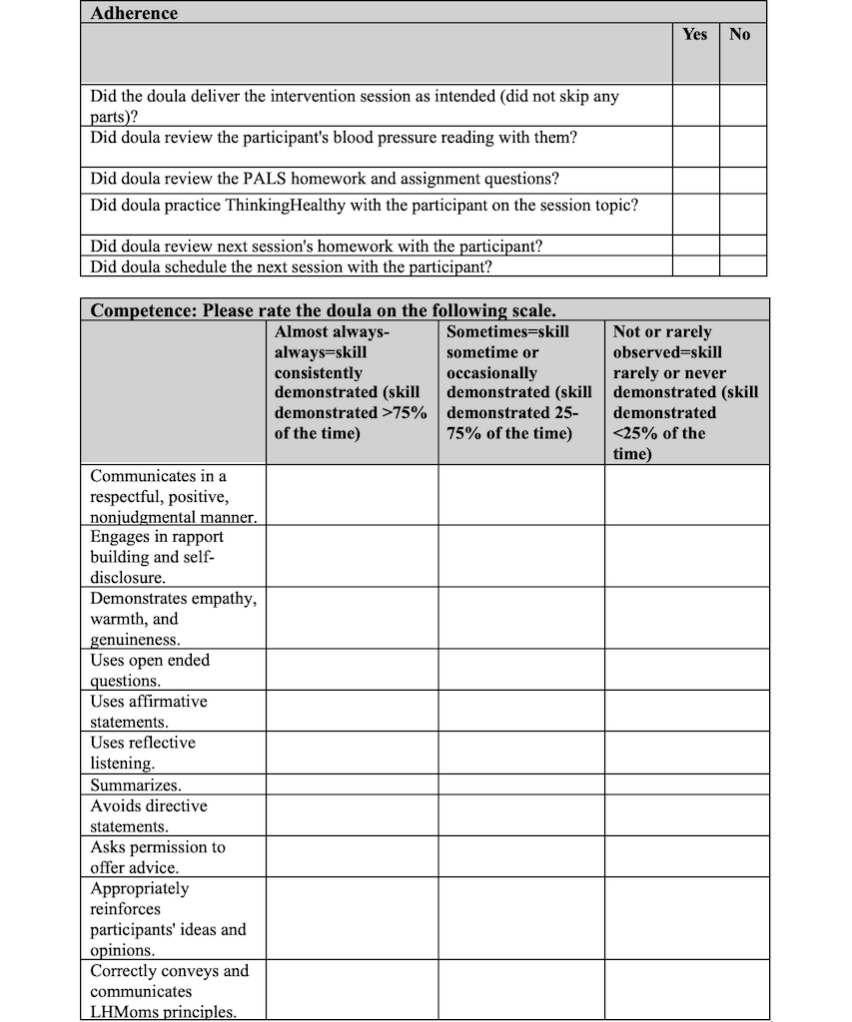
The Doula Fidelity Checklist assesses adherence and competence of doulas in delivering the LHMoms sessions. LHMoms: Living Healthy for Moms; PALS: Patient-Activated Learning System.

Once doulas expressed confidence in their ability to deliver a session, they were certified on each session by a study clinician (psychiatrist or psychologist with expertise in cognitive behavioral interventions), assuring that a minimum level of competence had been achieved. Certification used a Doula Fidelity Checklist, shown below in Figure 2, which is a rating assessment to evaluate doula competency and adherence to protocol in delivering each session. A “minimum performance” checklist was developed within the Doula Fidelity Checklist, and doulas had to deliver each session to this study’s psychiatrist or psychologist to meet the Doula Fidelity Checklist minimum performance standards before becoming certified. Doulas who did not meet the minimum performance standards on their initial certification attempt were asked to engage in additional practice until they met the minimum performance standards.

### Screening Procedures and Eligibility Criteria

The inclusion criteria for doulas include affiliation with our community partners (CWHA or NMPP) and experience working with 10 or more clients in the catchment areas of our hospital sites. Doulas planning to move out of the area in the next year are not invited to participate.

Inclusion criteria for the RCT include birthing individuals aged 18 or older, having delivered a singleton live birth, having self-identified Black race or Latinx ethnicity, or being a Medicaid beneficiary. Exclusion criteria are birthing individuals younger than 18 years; unable to communicate in English, Spanish, or Haitian Creole; using other nonhospital doula services already; having a multifetal pregnancy, known major fetal anomaly, or stillbirth; being an active user of intravenous drugs; having known suicidal ideation with intent and plan or a known primary psychotic disorder; being on hemodialysis; having gestational age <24 weeks at delivery; and having plans to move out of the area within the next 6 months.

For the implementation evaluation cohort, doula and birthing individual criteria will be as above. For hospital staff, the only inclusion criteria will be employment at the hospital site, working on obstetrical wards, being aged 18 years or older, and being able to communicate in English, Spanish, or Haitian Creole. Staff who have no interaction with birthing parents are not invited to participate.

### Randomization and Informed Consent

At each of the 3 hospital sites, 200 participants will be enrolled and randomized, 100 per arm. The blinded study statistician creates the randomization design to randomize doulas and participants on an equal (1-to-1) basis. This study’s statistician, recruiters at each hospital, and study principal investigators remain blinded to randomization until the participant’s completion of the last study assessment, which is 6 months post hospital discharge. The randomization of participants occurs after the recruiter has obtained informed consent and completed in-hospital enrollment, before hospital discharge. The research program coordinator in the Weill Cornell Medicine Obstetrics and Gynecology Department is unblinded and executes the randomization of all RCT participants.

Doulas in both the intervention arm and attention control arm are assigned to the participants randomized to the respective arm, with no crossover, using block randomization, also designed by the blinded study statistician and executed by the unblinded research program coordinator. RCT participants are made aware of the arm to which they are randomized. Doulas and RCT participants are not blinded throughout this study, as both parties are inevitably aware of the program in which they are participating due to preparatory doula training and from session content.

After enrollment, while still hospitalized, all participants are given a home BP monitor and are taught how to take their BP using the device. All participants are asked to record BP values in a logbook provided by this study.

### Participant Groups

#### Intervention Arm

The LHMoms intervention arm receives the doula-delivered intervention focusing on MH and cardiovascular health. The intervention promptly begins within the first day post discharge, when the doula calls each participant to introduce herself. In the 1-7 days post discharge, LHMoms intervention participants receive daily calls and texts from their doulas. Doulas monitor participants’ BP readings, collected in a daily log, and scan for postpartum complications: infection, bleeding, stroke, hypertension, cardiomyopathy, and depression. After the first 7 days, participants begin the 12-session CBT-based intervention, delivered via phone or Zoom every 1-2 weeks (Table 1).

**Table 1:** After the initial 7-day emergency detection period, the LHMoms^a^ intervention is delivered via 12 Zoom sessions^b-e^.

Session	Topic^b-d^
1	Overview, introduction to healthy thinking, and heart health
2	Sleep for mom
3	Stress
4	Bonding with baby
5	Physical activity^e^
6	Life’s Essential 8: cholesterol, BP^f^, and smoking
7	Life’s Essential 8: diabetes
8	Healthy eating
9	Relationships, community, health buddies, and asking for help
10	Planning for the future, pregnancy as a window to future health, and health passport
11	Review past session topics as desired
12	Final session and send-off

^a^LHMoms: Living Healthy for Moms.

^b^Each session includes cognitive behavioral therapy and cardiovascular health content tailored to the topic.

^c^Patient-Activated Learning System readings and videos for the next session topic are assigned for each session. Participants are also instructed to monitor their blood pressure, practice replacing negative thoughts with positive thoughts, and work on their individualized goals.

^d^Doula care is provided at each session with wellness check-ins, connections to resources, and screenings for suicidal ideation and intimate partner violence as needed.

^e^Doula care for session 5 includes a discussion of the participant’s changing body and body image.

^f^BP: blood pressure.

#### Attention Control Arm

Participants in the attention control arm receive a variation of standard doula postpartum care. Participants are instructed in the hospital on how to measure and record their BP at home daily. They do not have daily contact with their doulas in the first 7 days post discharge. Following that, they begin the 12-session attention control sessions, delivered via phone or Zoom every 1-2 weeks (Table 2).

**Table 2:** Attention control sessions are delivered via 12 Zoom sessions.

Session	Topic
1	Birth experience and your body
2	Supporting mom or infant feeding support
3	Infant feeding support
4	Baby sleep
5	Human papillomavirus
6	Newborn care
7	Reproductive life cycle
8	Sudden infant death syndrome and smoking
9	Well-child care and immunizations
10	Maternal health
11	Review past session topics as desired
12	Final visit and long-term support plan

### Doula Supervision and Assessment

Intervention doulas have weekly supervision sessions with a study psychologist and the LHMoms research coordinator. These supervision sessions happen throughout the 24 months of the doulas’ participation at their respective RCT sites. These sessions help ensure that each doula is delivering the LHMoms intervention as intended. Doulas can ask this study’s psychologist or psychiatrist questions about specific participant cases, LHMoms intervention delivery, or other issues that may arise. Additionally, each month, 2 randomly sampled LHMoms sessions are audio-recorded for each doula, with the consent of the doula and participant. These sessions are evaluated using the Doula Fidelity Checklist and are evaluated by this study’s psychologist for additional factors: nonverbal and verbal communication, rapport, problem-solving, and more.

Attention control doulas also engage in weekly supervision sessions with the LHMoms study coordinator as well as leadership from the respective community organization, CWHA or NMPP, offering opportunities to ask questions about specific participant cases and other issues that may arise.

### Data Collection

Data are collected from doulas as well as participants. Doulas complete a pretrial implementation survey collecting information on their background and demographics, doula training, and their views on the NYC postpartum care landscape, as well as the feasibility and acceptability of the intervention.

**Figure 3:**
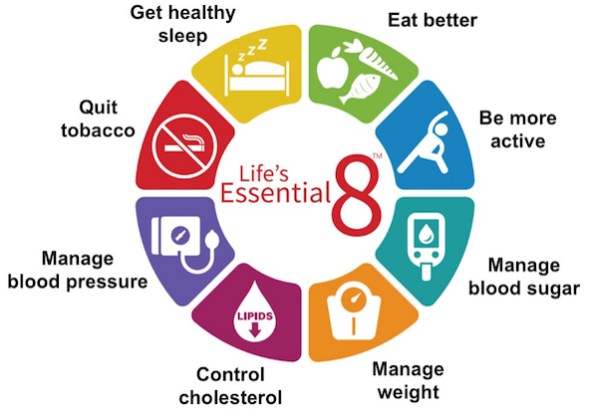
Life’s Essential 8 includes key areas for improving and maintaining cardiovascular health as defined by the American Heart Association.

For all trial participants, data are collected at baseline in the hospital predischarge, 2 weeks post discharge, 6 weeks post discharge, 3 months post discharge, and 6 months post discharge. The LHMoms study team collects data via web-based surveys on the primary outcomes of PPDS, using the Edinburgh Postnatal Depression Scale (EPDS) as a proxy for the incidence of symptoms of PPD (with scores >9 indicating the presence of PPDSs), and the “heart score,” an evaluation of LE8 provided by the AHA. Figure 3 below illustrates the key areas of LE8.

Quality of life is measured via short (15-minute) and long (30-minute) web-based surveys. Long web-based surveys, administered at baseline, 6 weeks, and 6 months, take 30 minutes to complete and assess psychosocial status and other health behaviors using the EPDS, General Anxiety Disorder Assessment, Pittsburgh Sleep Quality Index, Perceived Stress Scale, Brief Resilience Scale, Scale of Perceived Social Support, Posttraumatic Stress Disorder Checklist for *DSM-5* (*Diagnostic and Statistical Manual of Mental Disorders* [Fifth Edition]), and smoking status. Short web-based surveys, administered at 2 weeks and 3 months post discharge, take 15 minutes to complete and assess psychosocial status using EPDS, General Anxiety Disorder Assessment, and Pittsburgh Sleep Quality Index (Table 3).

**Table 3:** Data are collected via short and long REDCap^a^ surveys, and in-person measurements are taken by this study’s team^b-e^.

Data collection instruments and measures	Long web-based survey, 30-min^b^	Short web-based survey, 15-min^c^	In-person measurements^d^
EPDS^e^	✓	✓	
GAD-7^f^	✓	✓	
AHA^g^ My Life Check (Heart Score)^h^	✓		
PCL-5^i^	✓		
PSS-10^j^	✓		
PSQI^k^	✓	✓	
BRS^l^	✓		
Perceived social support	✓		
Hemoglobin A_1c_^m^			✓
Cholesterol			✓
Weight and height			✓
Blood pressure			✓
Smoking status	✓		

^a^REDCap: Research Electronic Data Capture.

^b^Baseline, 6 weeks, and 6 months.

^c^2 weeks and 3 months.

^d^Baseline, 3 months, and 6 months.

^e^EPDS: Edinburgh Postnatal Depression Scale.

^f^GAD-7: General Anxiety Disorder Assessment.

^g^AHA: American Heart Association.

^h^My Life Check is only completed in baseline and 6–month-long surveys.

^i^PCL-5: Posttraumatic Stress Disorder Checklist for DSM-5 (Diagnostic and Statistical Manual of Mental Disorders [Fifth Edition]).

^j^PSS-10: Perceived Stress Scale.

^k^PSQI: Pittsburgh Sleep Quality Index.

^l^BRS: Brief Resilience Scale.

^m^Hemoglobin A_1c_: glycated hemoglobin.

In addition to web-based survey administration, study staff collect physiological data at in-person study visits. Staff perform point-of-care testing for total and high-density lipoprotein cholesterol and hemoglobin A_1c_, and measure BP, height, and weight at baseline, 3 months, and 6 months. Point-of-care testing occurs in the hospital shortly after enrollment and at a community location at 3 and 6 months for the convenience of participants.

**Figure 4:**
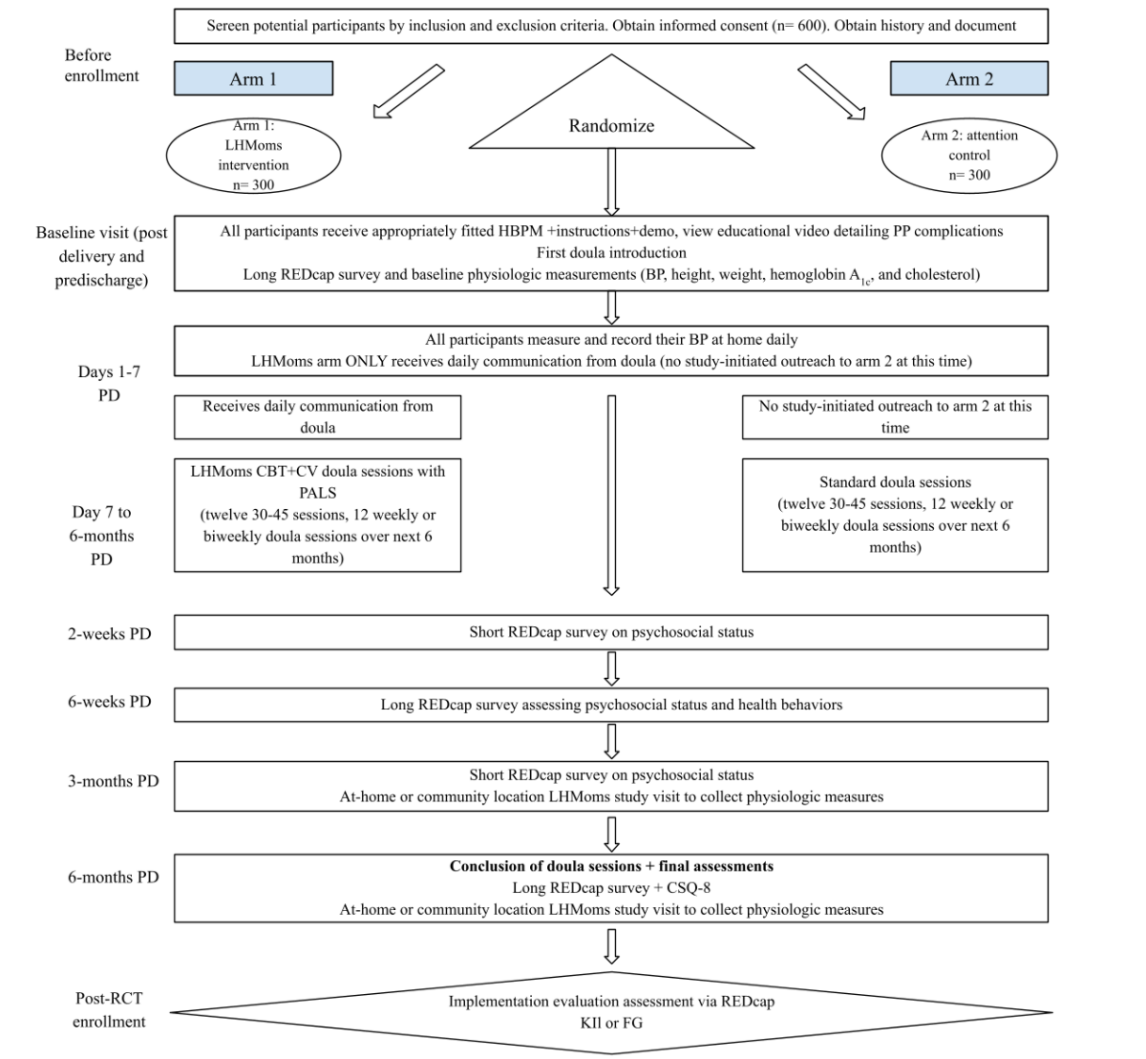
The RCT schema is shown below, beginning with participant enrollment in the hospital, postdelivery, and before discharge, and continuing until 6 months post discharge. BP: blood pressure; CBT: cognitive behavioral therapy; CSQ-8: Client Satisfaction Questionnaire-8; CV: cardiovascular; FG: focus group ; HBPM: home blood pressure monitoring; KII: key informant interview; LHMoms: Living Healthy for Moms; PALS: Patient-Activated Learning System; PD: post discharge; PP: postpartum; RCT: randomized controlled trial; REDCap: Research Electronic Data Capture.

Health care use is assessed via questions presented to all participants at 3- and 6-month post discharge regarding attendance of obstetric and primary care provider visits. This study’s team also performs an EHR review of clinical visits and encounters to understand contact with the individual health systems. A Client Satisfaction Questionnaire-8 (CSQ-8) is administered at 6 months. All data are collected in a secure REDCap database housed at the main study site. The RCT schema is shown below in Figure 4.

### Statistical Analysis: Analysis of the Dual Primary End Points

Preliminary statistical analysis examines baseline characteristics between study arms (LHMoms intervention vs attention control) to verify that the randomization generated comparable groups. Categorical variables are summarized as frequencies and proportions. Statistical significance is set at *P*<.05 and tested using chi-square or Fisher Exact tests for variables with low expected frequencies (eg, <5). Continuous variables are described as mean, SD, median, and IQR. Statistical significance is tested using a 2-tailed Student *t* test or Wilcoxon Rank Sum for variables with unequal variance or skewed distributions.

The primary outcome of PPDS is operationalized as the presence or absence of PPD symptoms as indexed by an EPDS score greater than or equal to 9 (EPDS >9) at 6 months post partum (Table 4). Linear mixed models, designed to account for the nonindependence of observations within the same person that is inherent to repeated measures designs, are used to estimate the mean EDPS score with 95% CIs and make group comparisons between the LHMoms intervention and attention control at baseline and 6 months post partum. This approach enables an intention-to-treat analysis whereby all participants randomized into the trial are included in the primary analysis regardless of the number of sessions completed due to the flexibility of linear mixed models to handle the presence of missing data. Baseline characteristics associated with PPDS in univariate regression models (eg, parity) or group imbalance detected at baseline are included in linear mixed models as relevant covariates.

**Table 4:** Primary and secondary outcomes measured in REDCap^a^ surveys assess psychosocial status and other health behaviors, as well as health care use and patient satisfaction.

Primary or secondary	Name of outcome	Specific measure to be used
Primary	PPDS^b^	EPDS^c^≥9
Primary	Cardiovascular health	LE8^d^ (continuous measure)
Secondary	Anxiety	GAD-7^e^
Secondary	Posttraumatic stress disorder	PCL-5^f^
Secondary	Perceived stress	PSS-10^g^
Secondary	Sleep	PSQI^h^
Secondary	Resilience	BRS^i^
Secondary	Social support	Perceived Social Support Scale
Secondary	Health care use	Contact at 7 days; 6 weeks postpartum visit attendance
Secondary	Satisfaction with postpartum care	CSQ-8^j^
Secondary	Implementation process	Focus groups, surveys, and interviews

^a^REDCap: Research Electronic Data Capture.

^b^PPDS: postpartum depressive symptom.

^c^EPDS: Edinburgh Postnatal Depression Scale.

^d^LE8: Life’s Essential 8.

^e^GAD-7: General Anxiety Disorder Assessment.

^f^PCL-5: Posttraumatic Stress Disorder Checklist for DSM-5 (Diagnostic and Statistical Manual of Mental Disorders [Fifth Edition]).

^g^PSS-10: Perceived Stress Scale.

^h^PSQI: Pittsburgh Sleep Quality Index.

^i^BRS: Brief Resilience Scale.

^j^CSQ-8: Client Satisfaction Questionnaire-8.

To determine changes in the second primary outcome of cardiovascular health, we similarly compare LE8 scores from baseline to 6 months with a linear mixed model approach as previously described. Following published guidelines, the LE8 metric is summed into a 1-100 score [17,45]. In addition to comparisons of mean scores at baseline and 6 months post partum, changes in group mean scores across this period are compared (ie, the interaction between treatment and time, eg, heart health score from baseline to 6 months post partum in the intervention vs attention control participants). Components of the LE8 score include diet, physical activity, tobacco use, sleep, weight, cholesterol, blood glucose, and BP—each of the 8 elements is scored separately, and a summary score is created. An 80 or higher on the LE8 metric is considered ideal, with 50-79 being intermediate, less than 50 meaning poor, with a range of 0 (poor) to 100 (ideal). Prior work shows a 10-point change is considered clinically meaningful [46].

For both primary end points, given the longitudinal nature of the intervention, dose is an important effect for exploration. Additionally, we model a site (ie, hospital) effect as a fixed main effect and explore a site by treatment interaction (eg, given the potential for personnel [ie, doula] differences across sites, there is potential for a site-level difference in treatment response).

### Sample Size Determination for Primary End Points

The EPDS scores from baseline to 6 months are used to analyze PPDS in LHMoms and control arms. While we considered PPDS and LE8 as dual primary end points, we chose 1 outcome to base our sample size on, which was PPDSs as defined by an EPDS score >9. This score was specifically chosen because it is used as the screening cutoff to refer for clinical evaluation in our hospital system, as it represents the possible presence of depressive or anxiety symptoms. The rate of EPDS >9 ranges from about 30% to 40% depending on other characteristics of the clinics (we selected 33.5% as the lower tertile of this observed range for our nonintervention group in the power calculation). We project we will observe a reduction in the PPDS proportion from 33.5% in the control arm to 23% in the intervention arm. This yields a minimum detectable difference of 31.5% lower incidence of PPDS in the LHMoms intervention arm compared to the attention control arm at 6 months. Our minimum detectable difference is calculated with a sample size of approximately 450 individuals (225 per study arm), assuming a 1:1 randomization, a design effect of 1.5 to account for variance among sites and doulas, a power of .90, a 2-sided significance level of 0.05, and a 20%-25% attrition. Our recruitment projection is for a sample of 600, 300 per arm; however, we have conservatively built in a 25% attrition rate for the longitudinal study (thus powering from a 450 analytic sample), although in past studies we have achieved 80%-85% retention in studies with a longer 1-year observation period [47-50].

### Analysis of Secondary End Points

The secondary end points include attendance at obstetric postpartum visits, linkage to primary care, and patient satisfaction (Table 4). The secondary end points are assessed through questionnaires at 3- and 6-month post discharge. The questions assess attendance of obstetric or primary care visits and an EHR review of clinical visits by this study’s team.

Participant satisfaction scores are measured using the CSQ-8 survey administered at 6 months post discharge as part of the long REDCap survey. The CSQ-8 survey consists of 8 questions, each weighing 4 points respectively; the survey consists of a range of responses, with 1 being “this does not apply to me” and 4 being “applies to me” [51]. Scores for the CSQ-8 survey can fall between 8 and 32, with higher scores reflecting greater satisfaction experienced by the participant with the care provided. For the CSQ-8 survey, scores 8-13 indicate poor satisfaction, 14-19 represent fair satisfaction, 20-25 mean good satisfaction, and 26-32 indicate excellent satisfaction. For our study, a 4-point difference on the CSQ-8 survey is defined as “clinically meaningful,” assuming an SD of 6 points [51]. No existing literature or research has a consensus on what point difference on the CSQ-8 survey can be considered “meaningful.” This is likely due to the CSQ-8 survey not being uniform and being applied in various settings. The estimated average for the CSQ-8 score for the control group is 13. This is due to the low satisfaction with care in the population that LHMoms is serving.

### Analysis of Tertiary End Points

The tertiary end points for this study evaluate the implementation outcomes and the process of implementing the LHMoms intervention. Tertiary end points evaluate the type 1 hybrid effectiveness-implementation trial using triangulated mixed methods data from interviews and surveys. Tertiary end points investigate the barriers to adopting the LHMoms program, doula competence and training, and strategies for implementation. Implementation strategies include RE-AIM and CFIR via EHR review and interviews or focus groups with doulas, clinical providers, hospital providers, and birthing individuals. CFIR consists of 5 domains—innovation, outer setting, inner setting, individuals, and implementation process (Table 5). CFIR is the “why,” as it helps guide the assessment of barriers and facilitators in the LHMoms implementation [52]. RE-AIM encompasses the “who, what, where, how, and when” as it examines implementing evidence-based interventions in real-world settings and how that implementation can be approved or adopted better (Table 6) [53].

**Table 5:** CFIR^a^ measures are applied to the LHMoms^b^ implementation evaluation.

CFIR construct		Example interview item	Example survey scale or item
**Innovation**			
	Relative advantage	What are the advantages of using LHMoms versus usual care? Versus other doula-based programs?	How essential is LHMoms to high-quality postpartum care? (Likert scale)
	Adaptability	How hard will it be to adapt LHMoms to your setting?	Adapted from previously used and validated scale items regarding relative advantage, complexity, cost, evidence, and trialability (Likert scale)
	Complexity	How hard will it be to implement LHMoms in your setting?	Adapted from previously used and validated scale items regarding relative advantage, complexity, cost, evidence, and trialability (Likert scale)
**Outer setting**			
	Local attitudes	How likely is this hospital to use LHMoms?	Level of enthusiasm at this hospital for this program (Likert scale)
	Partnership and connections	Do you have sufficient partnerships and connections to conduct LHMoms?	Level of existing partnerships (5-point Likert scale from “none” to “a lot”)
	Financing	Is there hospital-level funding for community programs?	Funding is a barrier for community programs (yes or no); ORC^c^ 4-item resources subscale
**Inner setting**			
	Relational connections	Are relationships with community partners well established enough to implement LHMoms?	I work with community doula organizations (5-point Likert scale from “none” to “a lot”)
	Communications	How hard will it be to implement communication between care providers or a hospital system and LHMoms?	Level of communication with organizations outside the hospital (5-point Likert scale from “easy” to “extremely difficult”)
	Tension for change	Do you think the current postpartum care system works well?	Current postpartum care is: 5-point Likert scale from “extremely inadequate” to “extremely high quality, no need for change”; ORC
	Relative priority	How important is fixing postpartum care compared to other needs?	Compared to other priorities, the importance of fixing postpartum care is: 5-point Likert scale from “not very important” to “the highest priority”
**Individuals**			
	High-level leaders	Is hospital leadership likely to be enthusiastic about the new program?	How likely are hospital leaders to embrace this program? 5-point Likert ORC 4-item leadership culture subscale
	Innovation deliverers	Will doulas embrace the new roles embedded within LHMoms?	How likely is it that doulas will embrace the enhanced roles required by LHMoms? 5-point Likert; ORC 4-item staff culture subscale
	Innovation recipients	Will patients such as LHMoms?	How likely is it that patients will find LHMoms acceptable? 5-point Likert scale
**Implementation process**			
	Teaming	Will it be challenging to form a team with community partners and hospital players?	How challenging will it be to form a team with community partners and hospital players? 5-point Likert scale from “not very” to “very”
	Tailoring	Will it be hard to find strategies to fit our context?	How difficult will it be to choose strategies to fit our context? 5-point Likert scale
	Adapting	Will it be hard to adapt LHMoms for your setting?	How difficult will it be to adapt LHMoms for this setting? 5-point Likert scale

^a^CFIR: Consolidated Framework for Implementation Research.

^b^LHMoms: Living Healthy for Moms.

^c^ORC: Organizational Readiness to Change.

**Table 6:** LHMoms^a^ implementation measures are guided by RE-AIM^b^.

RE-AIM construct	Description of construct	Outcomes	Phase
Reach	Who: what patients are intended to benefit, and who actually participates or is exposed to LHMoms?	Percent of patients approached who agree to participate Percent of participants who finish the program	Implementation
Effectiveness	What are the most important benefits you were trying to achieve, and what is the likelihood of negative outcomes?	Immediate postpartum outcomes: percentage of participants with indicated health care visit post partum Longer-term outcomes: Percentage of participants in each arm with PPDS^c^ Percentage of change in the number of LE8^d^ scores for participants in each arm Difference between arms in stress, resilience, and social support	Implementation and sustainment
Adoption	Where was LHMoms applied and by whom?	Number of doulas trained in LHMoms	Implementation
Implementation	How consistently was the program delivered, how was it adapted, how much did it cost, and why did the results come about?	Competency in LHMoms: number of trial sessions to be competent Treatment fidelity: mean observation score from a representative sample of audio-recorded sessions Barriers or facilitators: CFIR^e^ constructs	Preparation or implementation
Maintenance	When did LHMoms become operational; how long was it sustained; how long were individual results sustained?	Continued use of LHMoms: percentage of doulas who indicate they will continue to use LHMoms; percentage of intervention doulas who train other doulas Barriers or facilitators: CFIR constructs	Sustainment and ecologically valid sustainment

^a^LHMoms: Living Healthy for Moms.

^b^RE-AIM: Reach, Effectiveness, Adoption, Implementation, and Maintenance.

^c^PPDS: postpartum depressive symptom.

^d^LE8: Life’s Essential 8.

^e^CFIR: Consolidated Framework for Implementation Research.

### Ethical Considerations

#### Institutional Approval

This study was approved and reviewed by the Institutional Review Board (IRB) of Columbia University as the single IRB #AAAV1436. Weill Cornell Medicine and the individual hospital sites will rely on Columbia. This trial is registered on ClinicalTrials.gov (ID NCT06666400).

#### Consent Procedures

LHMoms study team members obtain written informed consent according to the International Council for Harmonisation, Good Clinical Practice, and local regulations. Oral explanations and written consent forms are available in English and will be available in Spanish and Haitian Creole. These consent procedures are IRB-approved and in compliance with the HIPAA (Health Insurance Portability and Accountability Act). Consent is documented via electronic signature on the IRB-approved REDCap consent form. Consent complies with Department of Health and Human Services regulations and describes the following principles of this study: purpose of this study, research procedures, foreseeable risks, benefits, alternative procedures, record and data storage details, compensation, information about who to contact with questions, and participation is always voluntary. Study staff obtaining informed consent acknowledge the possibility of a loss of confidentiality or privacy as a risk of study participation. Staff will assure participants that every effort will be made to maintain the confidentiality of their identity and data, as consent highlights deidentification of data and secure storage of deidentified data within a password-protected database or locked file cabinet only accessible to authorized staff. Compensation is explicitly outlined within the consent form, stating “all participants will receive compensation (in the form of *ClinCards*, a maximum of $200 compensation: $50 for the baseline visit, $35 for the first full data survey at 6 weeks, $20 for the shorter surveys completed at 2 weeks and 3 months, and $75 for the final survey at 6 months).” The currency is in US dollars.

### Care and Referrals

Participants are referred to appropriate treatment if they exhibit any symptoms of extreme psychological distress. For reference, if participants show signs of active suicidal ideation (including intent and plan), they are instructed to go to the emergency department and obtain immediate medical evaluation. If the participant cannot go to the emergency department, the doulas and study team members assist in calling 911. There are also protocols in place for participants reporting intimate partner violence and abuse, as well as urgent mental and physical issues. Participants are given contact numbers for clinical emergencies; these numbers are accessible 24/7. Participants are also referred to clinical and counseling resources and emergency care, as well as community-based resources (such as housing, food, and resources for intimate partner violence), as needed. Participants can access these resources through a link in the online surveys, and once accessed, principal investigators and coinvestigators follow up to ensure care is received correctly.

BP is monitored closely throughout RCT participation by the LHMoms study team and doulas. Specifically, the LHMoms BP safety protocol follows ACOG guidelines throughout the critical 7-day period and within the first 12 weeks postdelivery, and AHA guidelines thereafter. Participants are instructed to contact their obstetric provider, if within the first 12 weeks postdelivery, or their primary care provider, if more than 12 weeks postdelivery, for any mild or elevated range BP readings. Participants with severe or hypertensive crisis range BP readings with or without hypertensive symptoms are instructed to present to the emergency department immediately or call 911.

The LHMoms study team also monitors hemoglobin A_1c_ and lipids point-of-care testing results collected for research. In the event of an abnormal result, this study’s team instructs the participant to contact their primary care and obstetric providers. If baseline results are abnormal while the participant is still in the hospital, this study’s team also immediately alerts their hospital postpartum care team of their results.

The LHMoms study team and doulas provide any further assistance needed to connect participants to their care providers.

## Results

Data collection for participants from each of the 3 recruitment sites will be concluded by November 30, 2027, February 29, 2028, and August 31, 2029, respectively, for Brooklyn, Queens, and upper Manhattan. Thus, after completion of follow-up study activities for upper Manhattan participants, all data for the total expected 600 participants will be collected by the end of the grant year 6, August 31, 2029. The final grant year 7, from September 1, 2029, to August 31, 2030, will be designated for completion of data analysis. Data will be analyzed on an intention-to-treat basis by study team members blinded to participant conditions.

## Discussion

### Summary and Conclusions

This hybrid type 1 effectiveness- implementation RCT tests a novel nonspecialist intervention to prevent MH and cardiovascular health complications of childbirth. Our design is rigorous—we test the intervention against an attention control and measure numerous secondary outcomes having to do with health care use.

We hypothesize that, compared to attention control participants at 6 months post discharge, intervention arm participants will have at least 31.5% lower incidence of PPDS (score >9 on EPDS), and at least 1-point greater improvement in cardiovascular health using the LE8 score. We also expect that participants randomized to the intervention arm will experience better quality of life, reporting lower perceived stress and substance use and higher rates of resilience and perceived social support. Regarding health care use and satisfaction, we anticipate that more intervention than control individuals will have an indicated health care provider contact within the first 7 days post discharge or attend 6-week postpartum visits. We also hypothesize that intervention participants will report greater patient satisfaction with postpartum care, with higher scores on the CSQ-8 survey than control participants. We have culturally adapted an existing intervention to our local population in NYC, and will also be tracking specifics of implementation that will allow us to adapt the intervention further for other contexts. We will obtain important data from participants and key players in the implementation that will support and inform on the long-term sustainability and scalability of the LHMoms program.

### Dissemination

The LHMoms intervention is designed to reduce PPDS and cardiovascular complications during the postpartum period in pregnancy. Additional findings from the LHMoms intervention will help us understand how the LHMoms intervention may improve satisfaction with care within vulnerable populations. We expect that our findings will contribute to the literature on the use of nonspecialist interventions in low-resourced settings, as well as to the literature on doula care in the postpartum period.

## Appendix

Multimedia Appendix 1 Peer review report by the ZRG1 CTH-F (71) - Center for Scientific Review Special Emphasis Panel - Maternal Health Research Centers of Excellence (National Institutes of Health, USA).

## Abbreviations

ACOG: American College of Obstetricians and Gynecologists
AHA: American Heart Association
BP: blood pressure
CBT: cognitive behavioral therapy
CFIR: Consolidated Framework for Implementation Research
CSQ-8: Client Satisfaction Questionnaire-8
CVD: cardiovascular disease
CWHA: Caribbean Women’s Health Association
DSM-5: Diagnostic and Statistical Manual of Mental Disorders (Fifth Edition)
EHR: electronic health record
EPDS: Edinburgh Postnatal Depression Scale
GDM: gestational diabetes mellitus
hemoglobin A1c: glycated hemoglobin
HIPAA: Health Insurance Portability and Accountability Act
IRB: Institutional Review Board
LE8: Life’s Essential 8
LHMoms: Living Healthy for Moms
MH: mental health
MI: motivational interviewing
NMPP: Northern Manhattan Perinatal Partnership
NYC: New York City
NYP: NewYork-Presbyterian
PPD: postpartum depression
PPDS: postpartum depressive symptom
RCT: randomized controlled trial
RE-AIM: Reach, Effectiveness, Adoption, Implementation, and Maintenance
REDCap: Research Electronic Data Capture
T2DM: type 2 diabetes mellitus
THP: Thinking Healthy Programme

## Data Availability

As part of the National Institutes of Health Maternal Health Research Centers of Excellence Initiative, data will be shared with existing data repositories such as the National Institute of Child Health and Human Development Data and Specimen Hub. Before sharing, data science expertise will be used to maximize data comparability and facilitate data harmonization. The Innovation Hub will manage a secure, cloud-based data infrastructure and role-based access control for Research Center investigators in the Maternal Health Research Centers of Excellence initiative to access sensitive or deidentified data and develop documentation to ensure all datasets can be appropriately used by all Research Centers and the Implementation Science Hub. A data structure will be developed that will make the datasets accessible for controlled public use at the end of the program.
