# Supplementary material for: Doula-Delivered Cognitive Behavioral Training and Cardiovascular Health Intervention for Birthing Individuals in a Low-Income New York City Population: Protocol for a Living Healthy for Moms Randomized Type I Hybrid Effectiveness-Implementation Trial
Source: JMIR Res Protoc. 2025 Dec 10;14:e76871. doi: 10.2196/76871 (PMC12739457; doi:10.2196/76871)
Supplement: Multimedia Appendix 1 [file resprot_v14i1e76871_app1.pdf]

SUMMARY STATEMENT

PROGRAM CONTACT:
Caroline Signore
(301) 496-5577
signorec@mail.nih.gov

(Privileged Communication)

Release Date: 05/05/2023
Revised Date:

Application Number: 1 U54 HD113172-01
Formerly: 1U54MD018881-01

Principal Investigators (Listed Alphabetically):

BROWN, SEVONNA
DORVAL-MOLLER, MADELEINE
HALL, KELLI STIDHAM
OSBORNE, LAUREN M
REDDY, UMA (Contact)
(List continued at end of document)

Applicant Organization: COLUMBIA UNIVERSITY HEALTH SCIENCES

Review Group: ZRG1 CTH-F (71)
Center for Scientific Review Special Emphasis Panel
RFA-HD-23-035: Maternal Health Research Centers of Excellence

Meeting Date: 04/11/2023
Council: MAY 2023
Requested Start: 07/01/2023

RFA/PA: HD23-035
PCC: PPB -PS
Dual PCC: CPS12
Dual IC(s): MD, MH

Project Title: NY Community-Hospital-Academic Maternal Health Equity Partnerships (NY-CHAMP)
SRG Action: Impact Score:28
Next Steps: Visit https://grants.nih.gov/grants/next_steps.htm
Human Subjects: 30-Human subjects involved - Certified, no SRG concerns
Animal Subjects: 10-No live vertebrate animals involved for competing appl.
Gender: 1A-Both genders, scientifically acceptable
Minority: 1A-Minorities and non-minorities, scientifically acceptable
Age: 1A-Children, Adults, Older Adults, scientifically acceptable

Project Year	Direct Costs Requested	Estimated Total Cost
1	1,850,967	2,857,517
2	1,789,409	2,762,484
3	1,873,870	2,892,874
4	1,860,415	2,872,103
5	1,778,691	2,745,937
6	1,769,016	2,731,001
7	1,669,004	2,576,603
TOTAL	12,591,372	19,438,520

1U54HD113172-01 Reddy, Uma

RESUME AND SUMMARY OF DISCUSSION: This application proposes to develop the New York Community-Hospital-Academic Maternal Health Equity Partnership (NY-CHAMP) to shape the maternal healthcare ecosystem in New York City and the state of New York. The significance of the project is the focus on cardiovascular and mental health, both of which are leading causes of preventable maternal health conditions. The experienced project team has an existing strong foothold in the New York City and regional communities, which exhibit strikingly high rates of maternal morbidity and mortality, and a wide reach will be beneficial in implementing the project. The panel was also enthusiastic that the project has an emphasis on equitable work, a track record on training, and a clear plan on involving community and early career researchers. Innovations in the co-design for the equity framework, machine learning methods to predict risk of adverse maternal outcomes, use of mixed methods across project, and inclusion of doulas to deliver cognitive behavioral training are also strengths of the proposal. However, the reviewers expressed concerns with the investigative team and the approach. The reviewers considered having 9 multiple principal investigators (MPIs) as both a strength and a weakness. Including all stakeholders is a positive, but the proposal lacks details on how to coordinate the team, given that half of the MPIs are inexperienced in leading this type of complex center and that this is the first time that these partners are working together. The lead principal investigator and other members of the team are new to the city and region, and potential lag time for developing relationships with the community partners is not addressed. The approach does not include safety and policy issues related to abortion care, and sustainability of the interventions is not clear. Following discussion, the initial enthusiasm was dampened somewhat by the weaknesses. The panel concluded that the overall impact of the NY-CHAMP to improve maternal healthcare disparities is likely to be moderately high to high.

COMMUNITY PARTNERSHIP COMPONENT MERIT SCORE: 18
The applicants propose to develop the Community Engagement and Policy Action (CEPA) core to assist in re-shaping the maternal healthcare ecosystem in New York City and the state of New York. The CHARGE Board (Community-Hospital-Academic Research Governance) will be assembled to guide the community component. The panel was enthusiastic that the thoughtful plan emphasizes equitable work among the partners. In addition to the CHARGE Board, other strengths include the piloting of a community-led provider and patient training program and a co-design, parallel process in urban and rural sites to define hospital and neighborhood needs and gaps. The panel expressed a concern that the project team is very large, and a community members may not have enough protected time for the project. However, the reviewers remained enthusiastic that the overall impact of the community partnership component to improve maternal healthcare in New York City and New York state as likely to be high.

TRAINING COMPONENT MERIT SCORE: 19
The applicants propose to develop the Career Development and Training Core (CDTC) using an interdisciplinary and community-centered approach. The notable strengths of the CDTC are that it will serve as a regional career development hub and the focus on early stage investigators and investigators who are underrepresented minorities. A clear plan is proposed to emphasize on anti-racism, cultural, and inclusive structural training, and the project team has extensive experience in leading and implementing career development programs in maternal health disparities. Two academic institutions commit financial support for early stage investigators in this training component. A minor weakness is the lack of details in the mentor-mentee plan. Following discussion, the panel agreed that the overall impact of the training component in NY-CHAMP is likely to be high.

RESEARCH PROJECT 1 MERIT SCORE: 38

Research Project 1 proposes to develop a risk model for severe maternal morbidity that integrates three sources of data: electronic health records, Medicaid insurance claims, and social determinants of health (SDoH) data. The reviewers agreed that if successful, the ability to predict which patient will be at risk for adverse maternal health outcomes is highly significant. The model addresses both individual and population factors, which strengthen the proposal. Applying machine learning tools to generate the model and translating the findings through a clinical decision support tool are innovative methods. The large database of over 328,000 women in New York City, and the team's experience working with these types of databases are additional strengths. However, weaknesses in the approach were score driving for some reviewers. The aims were thought to be interdependent, and how population level data will be integrated lacks details. The feasibility of translating the data to the clinic is unclear because there is no current model for clinical decision support. There were concerns about statistical rigor and potential cross-contamination of data. After discussion, the opinions of the panel were divided. The overall impact of developing a risk model to improve maternal healthcare disparities is likely to be moderately high to moderate.

RESEARCH PROJECT 2 MERIT SCORE: 28

In Research Project 2, the applicants propose to determine the effectiveness of the two-part intervention Living Healthy for Moms (LHMoms), which will be implemented in Brooklyn, Queens, and northern Manhattan in New York City. During discussion, there was agreement that if successful, the intervention has the potential to advance the field in continuity of care for post-partum patients. The emphasis on cardiovascular and mental health conditions, which are highly prevalent in post-partum mothers, is a strength. Doulas are under-utilized in the community, and the panel expressed enthusiasm over the novel concept of training doulas to serve as the lead in an emergency detection program. For some reviewers, however, weaknesses in the approach were score driving. The 12-week trial period was felt to be too short to enable refinement of the method. The mechanism for selecting doulas to participate in the trial is not included, and confidentiality concerns with doulas who are recruited from the community are not addressed. Several intervention components will be evaluated, and methods to determine the direct cause and effect are not included. After discussion, the reviewers remained mixed in the weighting of strengths and weaknesses. The majority of the panel concluded that the overall impact of the project to improve post-partum maternal healthcare is moderately high.

RESEARCH PROJECT 3 MERIT SCORE: 26

In Project 3, the applicants propose a mixed methods approach to determine the role of social and structural racism in maternal health outcomes. The reviewers noted that the project is timely in examining the effects of policies, costs, and hospitals, which have not been investigated together in depth. If successful, the high significance of the project lies in providing data to support evidence-based policy changes to address the effects of race at the multiple levels. The investigators, especially the inclusion of a community principal investigator, and the team's expertise are major strengths. The combination of qualitative and quantitative data in examining multi-level systemic racism is an innovative approach to inform policy changes. The proposed establishment of a research database for 10 million childbirths in 21 states will be a valuable resource. For some reviewers, the generalizability of the study on a national scale is unclear, given the focus on data from New York City. A detailed method of linking the qualitative and quantitative data is lacking, and interdependence of structural racial measures was a concern. Following discussion, the majority of the reviewers agreed that the weaknesses are minor, and the overall impact of Project 3 to improve maternal healthcare disparities is high.

DESCRIPTION (provided by applicant): The broad goal of the New York Community-Hospital-Academic Maternal Health Equity Partnerships (NY- CHAMP) is to upend the maternal healthcare ecosystem in New York City (NYC) and New York State (NYS) by establishing a sustainable, highly

collaborative infrastructure for community-centered maternal health science. We have carefully co-designed, with our partners, a research, training, and community engagement agenda that will 1) identify and address the comorbid biologic and psychologic pathways linking adverse social determinants of health (SDoH) to disparities in SMM and MM; and 2) co-create and implement scalable multi-level strategies grounded in anti-racism and empowerment to inform systems and policy change in our city and region. NY- CHAMP is set against the compelling backdrop of persistently high rates and disparities in SMM/MM across urban and rural parts of our state alike, despite a multitude of healthcare facilities and resources. More effective partnerships that break down siloes and leverage the knowledge and strengths of our diverse communities, hospitals, academic institutions, and government agencies are essential to assuring equitable access to culturally congruent, patient-centered care, redressing SMM/MM inequities, and maximizing our collective impact. We convene Columbia University and Weill Cornell Medicine, four lead community organizations (Black Women's Blueprint, Caribbean Women's Health Association, Northern Manhattan Perinatal Partnership, and The Bridge), and a broad coalition of multisector actors to address 3 synergistic Overall Aims. In Aim 1, we will implement a unified, community-driven research program that will: Project 1) Develop and implement novel artificial intelligence risk prediction models and a resulting clinical decision support system for SMM, accounting for SDoH, bias and fairness. Project 2) Conduct a Type 1 Hybrid Effectiveness-Implementation Trial of a novel doula-delivered cognitive behavioral therapy program holistically designed to address CV, MH, and SDoH outcomes during postpartum; build trauma-informed and empowerment-based service delivery models; and strengthen hospital-community care linkages. Project 3) Apply mixed methods to evaluate the effects of and excess costs attributable to structural racism and SMM/MM and the mitigating effects of two policy interventions for reducing maternal health disparities. In Aim 2, we will establish the Community Engagement and Policy Action Core (CEPA) to serve as an enduring platform for iterative community input, co-design processes, and research dissemination, capacity building, and policy action. In Aim 3, we will establish the Career Development and Training Core (CDTC) to transform the maternal health research culture and pipeline of early-stage investigators expertly trained in community partnerships, interdisciplinarity, anti-racism, and diversity, equity, inclusion, and justice. Our vision is perfectly aligned with the priorities, needs, and assets of our communities and with the NIH's IMPROVE initiative. Ultimately, NY-CHAMP will alter the underlying structural drivers of SMM/MM in NYC and NYS and shift the scientific paradigm for excellence in maternal health research.

COMMUNITY PARTNERSHIP COMPONENT PROJECT SUMMARY: **NY-CHAMP Community Engagement & Policy Action Core (Description as provided by applicant):** The NY-CHAMP Community Engagement and Policy Action Core (CEPA) will bring together an interdisciplinary, multi-sector team in a shared leadership and governance model to direct our proposed center of excellence. We convene for the first time four esteemed community multiple principal investigators (MPIs) - Black Women's Blueprint (BWB), Northern Manhattan Perinatal Partnership (NMPP), Caribbean Women's Association (CWHA), and The Bridge Directory, with a broader coalition of community organizations, hospitals, academic institutions, and governmental agencies from across NYC and NYS deeply invested in maternal health. The broad goals of our NY-CHAMP CEPA are to assure 1) local community needs, priorities, and assets are the very center of our Research Center of Excellence, and 2) our scientific endeavors result in maximum impact as well as structural and policy change in ways that fundamentally alter the drivers of health and health equity for birthing people in NYS and NYS. To achieve these goals, CEPA will practice intentionality, reflexivity, humility, and trust-building to lead four aims that serve as critical foundation and scaffolding for NY-CHAMP's community engagement efforts. In Aim 1, we will provide a sustained platform for iterative community input and engagement across our research center through the Community-Hospital-Academic Research Governance (CHARGE) Board. In Aim 2, we will launch a novel community-led maternal health provider + birthing patient training program that prepares hospital actors for anti-racism, cultural humility, implicit bias, and racial allyship

care; and prepares birthing patients for empowerment, self-advocacy, and activation. In Aim 3, we will conduct a locally led, co-design process in parallel urban and rural sites with two safety-net hospitals of the NYC Health + Hospitals network and in two counties in upstate New York in partnership with the University of Rochester and NYS Department of Health, to set the stage for scalability for our interventions, findings, and partnership model. In Aim 4, we will facilitate community-engaged research dissemination, collaboration, and policy action for maternal health equity in NYC and NYS, including a novel community-led research capacity building project for an innovative virtual doula care delivery platform. These aims, and the NY-CHAMP goals they support, are perfectly aligned with the NIH's IMPROVE and UNITE Initiatives to solve and resolve the maternal mortality and morbidity crisis and reduce disparities for birthing people and families across NYC, NYS, and ultimately the nation.

TRAINING COMPONENT PROJECT SUMMARY: **Career Development and Training Core (Description as provided by applicant):** The NY-CHAMP Career Development and Training Core (CDTC) will serve as a local and regional scientific career development hub that 1) offers resources and activities to foster cross-disciplinary, cross-sector collaboration and 2) sets the stage for a highly diverse, highly collaborative network of scientists poised to advance innovative, community-centered, anti-racist, multi-level solutions for maternal health equity. The CDTC will draw on the rich institutional resources of Columbia and Cornell University Medical Centers and synergize with the vast knowledge and assets of our strong community, governmental, hospital, and academic partners to transform the pipeline of maternal health scientists to be interdisciplinary in approach and committed to community-centered, health equity, and anti-racism research. In Aim 1, we will transform the maternal health research culture to create a pipeline of diverse scholars who are committed to community engagement and expert in interdisciplinary, multi-sector research partnerships. In Aim 2, we will operate as a hub for maternal- health-focused training opportunities and resources in structural racism, cultural humility, racial allyship, and diversity, equity, inclusion, and justice. In Aim 3, we will engage early-stage investigator (ESI) NY-CHAMP Scholars in hands-on training in intervention science that is community-led, anti-racist, and interdisciplinary via a collaborative, to-be-designed demonstration project. Our training philosophy values co-creation and co- implementation of methods and interventions informed by, respectful of, and responsive to the needs of the diverse communities we serve. The CDTC will contribute to the overall goals of the NY-CHAMP Center of Excellence by grounding scholars in the culture and structural change that is required to foster innovative new science and sustained community partnerships for research impact on maternal heath equity from the next generation of researchers.

RESEARCH PROJECT 1 PROJECT SUMMARY: **Using EHRs, Claims and SDoH data to Develop and Implement AI Risk Models for Predicting Maternal Morbidity and Healthcare Utilization (Description as provided by applicant):** Severe maternal morbidity (SMM) is defined as an unexpected adverse outcome during pregnancy or postpartum (PP) with significant short- or long-term negative consequences to a woman's health. Rates of SMM have risen substantially in the past decades and are >50% higher for non-Hispanic Black women than non- Hispanic White women. Leading causes of SMM and associated deaths are preventable and result, in part, from a systems-level failure to recognize and manage individual-level co-occurring physical, mental, and social risk factors that women experience across the perinatal, intrapartum, and PP periods. Adequate prenatal care and appropriate management of chronic conditions during pregnancy through a multilevel perspective may comprehensively mitigate rising maternal risk. However, important knowledge gaps limit efforts to improve and optimize care. First, clinicians and health systems are limited in predicting: 1) patients who will be most likely to experience inadequate prenatal care; and 2) patients who will have complications such as SMM. Second, social determinants of health (SDoH), defined as, conditions in the places where people live, learn, work, and play that affect a wide range of health and quality-of life-

risks and outcomes, may be an important determinant of outcome disparities and inadequate prenatal care. However, SDoH may be suboptimally captured by using routine screening questions during patient encounters and population-level exposures. We propose addressing these critical knowledge gaps by leveraging highly granular, longitudinal clinical data linked with SDoH data derived from the Patient-Centered Outcomes Research Institute (PCORI)-funded INSIGHT Clinical Research Network (Project Lead Pathak) on >328,000 women across New York City (NYC). In Aim 1, we will link longitudinal EHRs and claims data and characterize the study cohort. In Aim 2, we will ascertain community- and individual-level measures of SDoH by linking population-level SDoH data for the study cohort using natural language processing (NLP) and machine learning (ML) methods from clinical note narratives. In Aim 3, we will predict the risk of inadequate prenatal care and SMM, as well as assess fairness and bias through ML models. Our rich multi-level data, measurement, and analytical approach will apply ML models to analyze electronic health records (EHRs), health insurance claims, and community/neighborhood- and individual-level SDoH data. In Aim 4, we will assess the usability and acceptability of a pilot integration of risk models into point-of-care clinical decision support (CDS) at two study sites (Weill Cornell Medicine and Columbia University Irving Medical Center) using patient and community input to inform development and use. Ultimately, this project advances the overall goals of NY- CHAMP to optimize risk prediction of inadequate prenatal care and adverse maternal outcomes, including SMM.

<u>RESEARCH PROJECT 2 PROJECT SUMMARY:</u> **Living Healthy for Moms (Description as provided by applicant):** Most preventable maternal deaths occur in the intrapartum and immediate postpartum (PP) periods, as do complications from undetected/undertreated mental health (MH) conditions (e.g., suicide, overdose) and cardiovascular (CV) events. In New York City (NYC), maternal deaths are 8–12 times higher for Black than for White birthing parents. However, most community-based programs addressing the health of birthing individuals in this population have been narrowly focused on basic services (e.g., breastfeeding, social support), with limited focus on evidence-based care. Holistic interventions addressing physical, mental, and social health needs in the critical PP transition and moving from trauma- and “risk”-based to a strength- and empowerment-based approach are urgently needed. Many hospital, community, and governmental barriers preclude broad adoption and scaling of doula-delivered care, and it is not known how facilitators, assets, and resources can improve care continuity and community-hospital linkages to support birthing individuals at diverse sites across NYC. The impact of doula- led interventions at patient- and health-system levels have yet to be rigorously evaluated. In collaboration with the Caribbean Women's Health Association and the Northern Manhattan Perinatal Partnership, we will address these gaps by implementing and evaluating Living Healthy for Moms (LHMoms) in three complementary settings and populations (Brooklyn, Queens, and Northern Manhattan). LHMoms is a novel integrated care intervention that focuses intensively on care continuity and community-to-healthcare linkages that starts prior to discharge and during the first 7 days post-discharge (PD) and extends into six months post-partum, as critical windows to prevent long-term physical and mental health sequelae, while also addressing key determinants of lifelong health risk. We have developed several innovative, evidence-based interventions, including an online education empowerment program (using a Patient Activated Learning System platform we developed) to build patient self- advocacy beginning in-hospital; a doula-led health emergency detection program during the critical first 7 days PD; and a trauma-informed doula-delivered cognitive behavioral phone intervention to address PP depression and cardiovascular risks for the critical six months following delivery. We propose a rigorous, mixed-methods study of LHMoms to address three specific aims. In Aim 1, we will test the effectiveness of LHMoms vs. attention control in a Hybrid Type 1 Implementation Trial with 450 randomized birthing individuals at three hospital sites in Brooklyn, Queens, and Northern Manhattan. This will include refining and tailoring the intervention to each site to maximize care quality, building capacity for doulas to deliver LHMoms, and assessing effectiveness in lowering PP depression, improving CV health, and creating better quality of life. In Aim 2, we will analyze the effects

of LHMoms on PP healthcare utilization and satisfaction. In Aim 3, we will examine the implementation process and outcomes of the LHMoms intervention at the three sites. This project will advance the overall goals of NY-CHAMP to optimize PP outcomes for historically disadvantaged birthing individuals.

RESEARCH PROJECT 3 PROJECT SUMMARY: **Policy solutions for addressing structural racism in maternal health disparities (Description as provided by applicant):** The broad, long-term objective of NY-CHAMP's Project 3 is to inform efforts to improve population maternal health equity by understanding and addressing the underlying determinants of racial and ethnic disparities in severe maternal morbidity (SMM) and mortality (MM) and ensuring equitable access to quality, culturally appropriate maternal health care. U.S. racial and ethnic minoritized birthing people are about three times as likely as non-Hispanic White birthing people to experience life-threatening complications and die during pregnancy and postpartum. Persistent racial and ethnic disparities in maternal health are not fully explained by known risk factors. Although structural racism (SR) is viewed as the fundamental cause of health disparities generally, few, if any, studies have empirically evaluated it role in excess SMM and mortality among racial and ethnic minority birthing people. Evidence regarding effective policy interventions—at state, local and hospital levels—to address SR in SMM/MM is even more scant. We propose a rigorous mixed methods project with specific aims to: 1) assess the associations of multidimensional indexes of SR with racial and ethnic disparities in SMM, suicidality, and mortality; and estimate the hospital costs of SMM attributable to SR; 2) assess the associations of two policy-relevant interventions (i.e., Medicaid eligibility generosity and healthcare workforce diversity) with racial and ethnic disparities in SMM, suicidality, and mortality; and quantify the moderating effects of these two interventions on associations between SR and maternal health outcomes; and 3) analyze the lived experiences of patients and community and hospital stakeholders to identify the mechanisms of, and effective solutions for, SR in maternal health care. We will analyze a robust triangulated set of quantitative and qualitative data from multiple sources, capturing both national and local samples, and apply a complementary suite of multilevel modeling, causal inference, and grounded theory research techniques. Data from the Healthcare Cost and Utilization Project, which provides information on 10 million childbirths from over 1200 hospitals in 21 states during 2016-2022 (Aims 1-2), will be complemented by prospective in-depth interview and focus group data from multisector stakeholders (patients, community, hospital) in three New York City hospital sites (Brooklyn, Washington Heights, Queens) of focus for NY-CHAMP's Intervention Project 2. Aims 1 and 2 will test the hypotheses that: 1) multidimensional indexes of SR are associated with significantly increased disparities in SMM, suicidality, and mortality; 2) higher state Medicaid eligibility generosity and healthcare workforce diversity are independently associated with lower disparities in SMM, suicidality, and mortality; and 3) Medicaid eligibility generosity and healthcare workforce diversity independently mitigate the impact of SR on disparities in maternal outcomes. Aim 3 will be hypothesis generating. Findings will help close important gaps in our understanding of the role that SR plays in SMM/MM disparities and generate urgently needed evidence for informing multilevel policy interventions to improve maternal health equity in our city, state, and across the country.

PUBLIC HEALTH RELEVANCE: The broad goal of the New York Community-Hospital-Academic Maternal Health Equity Partnerships (NY- CHAMP) is to launch a novel community-centered research, training, and engagement program that will identify and address the comorbid biologic and psychologic pathways linking adverse social determinants of health to disparities in severe maternal morbidity and mortality (SMM/MM); and with our partners, co-design scalable multi-level strategies grounded in anti-racism and empowerment to reduce SMM/MM and affect systems and policy change for maternal health equity in New York City and State. NY-CHAMP brings together Columbia University, Weill Cornell Medicine, four lead community organizations, and a broad coalition of community, hospital, academic, and government partners in a highly collaborative, paradigm-shifting model for excellence in maternal health research.

CRITIQUE 1:

Significance: 2
Investigator(s): 4
Innovation: 2
Approach: 4
Environment: 1
Community Partnership Component: 2
Training Component: 2
Research Project 1: 2
Research Project 2: 3
Research Project 3: 3

Overall Impact: The NY-CHAMP proposal is led by an exceptional team and environment that includes strong academic and community leadership, attention to equitable partnerships and community co-design. The center proposal integrates several methodological innovations and conceptual frameworks that are woven throughout study aims/projects with a focus on multilevel strategies to improve maternal health disparities. The research projects are innovative and complementary, spanning risk prediction using innovative AI and natural language processing methods to doula delivered behavioral interventions and an evaluation of several potential policy impacts. The proposal has a clear plan for involving community stakeholders and supporting underrepresented minority and early career researchers. Minor weaknesses include a large leadership team (9 MPIs), with some relatively junior community MPIs with limited/no research experience; a well-established PI Dr. Reddy with extensive experience managing large and complex research projects over the past 20 years but who is new to the primary institution thus may reflect less established community partnerships. This is the first time bringing together these different organizations/partners, so clear communication plans are needed across 9 MPIs in both academic and community settings. Lack of consideration of in-person meetings for such a large leadership team is a minor weakness. There was also overlap across some stakeholder/advisory groups that could be further specified. For research findings, pragmatically how findings would inform each other is not clear, including how P1 risk prediction results would feed into doula PP emergency detection program in P2 and how P2 qual data would inform P3 data collection. However, this does not detract from an excellent application with high potential for impact given the novel conceptual and methodological frameworks, co-design by community partners, and strong leadership team.

1. Significance:

Strengths

- NY state and NYC both have very high rates of maternal morbidity and mortality, particularly among Black women.
- Mental health and cardiovascular conditions are two leading causes of preventable maternal health, disability and disparities in NY and are the primary focus of this center.
- Multilevel approaches that incorporate structural factors and engage stakeholders in collaborative research has high potential for impacting system-level and policy change; risk prediction, intervention & policy solutions are the main aims of each of the research projects.
- Explicit focus throughout the proposal on health equity, DEI priorities, and promoting bidirectional partnerships.

Weaknesses

- PI also has a maternal fetal medicine research network pending.

2. Investigator(s):

Strengths

- NY CHAMP investigators have extensive previous experience in maternal health and health equity. Team has been engaged in almost every initiative by NYC and NYS to address SMM/MM.
- The team has a long history of leading NIH-funded research that has informed this project and strong history of collaboration across academic PIs (Reddy, Hall).
- Contact PI Dr. Reddy is the Director of the Division of Research and Innovation and has extensive experience managing large and complex research projects over the past 20 years and has 80% protected time for research.
- Leadership team spans academic MPIs across multidisciplinary units at Columbia/Cornell as well as leads from four community organizations. Inclusion of community-based organizations as part of leadership team is a strength and demonstrates commitment to equity.
- Focus on training infused throughout, including pairing established/senior investigators with early-stage investigators and community leaders.
- Strong track record of involvement in NIH training programs (Reddy, Hall). Hall has experience developing and leading a Center focused on maternal health disparities.
- Experience with Medical data and other data linkage expertise (Safford).
- The nine MPIs have distributed and shared leadership across all the research projects and cores which leverages their specific expertise and ensures the integration of all center components.
- MPI Hall has experience developing and leading a Center for Reproductive Health Research in the SouthEast (RISE) – a scientific hub at Emory University that engages community partners and diverse stakeholders in rigorous evaluations of policies impacting access to care, service delivery, and outcomes for childbearing women, as well as in policy/advocacy action and research infrastructure and capacity building across the SE region.

Weaknesses

- The MPI team is quite large (9 MPIs). Further, it is the first time bringing together these groups so clear plan for communication is essential across 9 MPIs in both academic and community settings. Communication plan indicates that “MPIs are located at different sites throughout New York City, they will communicate mainly through calls and email; however, should the need arise to meet in person, they are a short taxi or subway ride from one another.” The lack of in person meetings given such a large leadership, diverse team may interfere with effective communication and collaboration throughout the project.
- Including community partner leaders as MPIs is a strength and demonstrates commitment to equitable partnerships. However, some community MPIs have limited/no prior research experience, and some are relatively junior (St. Clair), a clear plan for supporting community partner MPIs in research leadership roles is needed.
- Similarly including ESIs (e.g., Osborne) in the MPI team is a strength and demonstrates commitment to training, but given still on a K23, a clear plan for supporting their career trajectory while taking on this type of role is essential.
- PI Reddy is extremely qualified to lead this Center, however, worth noting that she only recently joined Columbia (January 2022); thus, community partnerships may still be nascent. Yet she

does also have a long history and connection to the New York communities this Center aims to serve. Other MPIs also have new/ pending appointments at Columbia (Osborne).

- PI Reddy has 80% protected time for research but is already PI for 5 large grants at Columbia and co-I for 5 additional grants, thus ensuring sufficient time to lead this Center, along with sufficient time for other co-Is and community partner MPIs will be essential.
- There are some overlapping roles across the ELC, steering committee, CHARGE Board, and internal leadership advisory board and external scientific advisory board.
- Unclear if MPI Hall's former Center for Reproductive Health Research in the SouthEast (RISE) was sustained.

3. Innovation:

Strengths

- Use of a co-designing for equity framework, and co-design with community partners across urban and rural sites is innovative.
- Use of AI/machine learning to predict individuals at higher risk for adverse maternal outcomes using natural language processing in EHRs is novel (PI). Research projects span AI prediction tools with a translation focus on policy impact.
- Innovative use of mixed methods across projects. P3 combines national quantitative data capturing 10 million childbirths in 21 states with local interview and focus group data capturing the lived experiences and perspectives of patients and community/hospital stakeholders in the same 3 diverse NYC hospital sites as P2.
- Focus on training doulas to deliver CBT is novel (P2).

Weaknesses

- The inclusion of many intervention strategies in Project 2 may not allow for understanding of which intervention components are most innovative/impactful.

4. Approach:

Strengths

- Multi-level framework guides all research, training, and engagement efforts.
- Focus on sustainability, collaboration, and equitable, bidirectional community partnerships throughout Center proposal. Focus on sustainability with partnership from the start.
- Center aims and projects were co-designed with community partners and focus on both rural and urban populations.
- Propose multilevel approaches that also incorporated lived experiences and equitable community partnerships.
- Rigorous methods include AI risk tools, implication science designs, task sharing with doulas, and innovative mixed methods (P3).
- Focus on unique challenges of URM in CDTC a strength – and prioritization of community partnerships and focus on community partnerships in pilot awards.

Weaknesses

- Given the range of intervention components evaluated in P2 it is possible design will not be able to disentangle which component is driving any effects.
- How findings would inform subsequent projects is not entirely clear – including how P1 risk prediction would feed into doula PP emergency detection program in P2 and how P2 qual data would inform P3 data.

- Doula interventions are largely online without opportunities to explore in person needs which may be relevant in underserved populations.
- Given the extensive intervention components of LHM intervention (Project 2), how the community partners would sustain this intervention without grant funding is not clear.
- Process or feedback from co-design process in developing the protocol is not clearly specified.

5. Environment:

Strengths

- Columbia and Weill Cornell in collaboration with four leading community organizations (Black Women's Blueprint, Caribbean Women's Health Association, Northern Manhattan Perinatal Partnership, and The Bridge) provide an excellent environment to conduct this work.
- NYC Dept. of Health and Mental Hygiene (DOHMH)-funded Maternal Hospital Quality Improvement Network (MHQIN) involves 14 of the 38 maternity hospitals including 8 NYC Health + Hospitals (H+H), a large safety-net system which serves one-third of all births in NYC. NY-CHAMP will build upon MHQIN, which supports SMM data collection and review as well as integration of doulas and midwives into existing maternity care structures at their participating hospitals.
- Strong institutional commitment, including contributions from Columbia and Cornell for pilot award programming, and strong institutional commitment and resources for DEI efforts.

Weaknesses

- First time bringing together these groups so clear plan for integrating teams across academic and community environments is essential.

Study Timeline:

Strengths

- Project 2 - Doulas have already been selected suggesting feasibility of startup.

Weaknesses

- Project 1 - concerns regarding timeline for Aim 4 clinical implementation if there are delays in Aims 1-3 leading to the development of the tool.
- Project 2 - One year may not offer sufficient time to refine the intervention.

Protections for Human Subjects:

Acceptable Risks and Adequate Protections

Data and Safety Monitoring Plan (Applicable for Clinical Trials Only):

Acceptable

Inclusion Plans:

- Sex/Gender: Distribution justified scientifically
- Race/Ethnicity: Distribution justified scientifically
- For NIH-Defined Phase III trials, Plans for valid design and analysis: Not applicable
- Inclusion/Exclusion Based on Age: Distribution justified scientifically

Vertebrate Animals:
Not Applicable (No Vertebrate Animals)

Biohazards:
Not Applicable (No Biohazards)

Resource Sharing Plans:
Acceptable

Authentication of Key Biological and/or Chemical Resources:
Not Applicable (No Relevant Resources)

Budget and Period of Support:
Recommend as Requested

Community Partnership Component
Principal Investigator(s): Hall, Kelli Stidham

Impact: The NY-CHAMP Community Engagement and Policy Action Core (CEPA) aims to ensure that the local community's needs and priorities are at the center of the Center and to maximize impact of the research projects in terms of structural and policy change to promote health equity for birthing people in NY. Strengths are that leadership includes 4 MPIs from partner community organizations. The CEPA core includes developing a governance board (CHARGE) board to be a platform for iterative community input and engagement which consists of an18-member Community-Hospital- Academic Research Governance (CHARGE) Board representing diverse, complementary community organizations, hospital partners, government entities, and academic institutions from NYC and NYS. The core also proposes launching a novel community-led maternal health provider + birthing patient training program that includes a focus on structural racism. The CEPA will guide a co-design process in parallel urban and rural sites to promote scalability of interventions, as well as have a novel research capacity building project with an innovative virtual doula care delivery program (Poppyseed Health), which are all innovative, impactful contributions of this community core. Minor score-driving weakness includes 1) some overlap across CHARGE board/Center leadership and ensuring sufficient protected time of Center leadership and community partners for engagement; 2) questions regarding the timeline (whether having the trainings, parallel co-design and Poppy seed demonstration project focus only in Years 6-7 will be feasible.

Training Component
Principal Investigator(s): Taylor, Jacquelyn

Impact: The NY-CHAMP Career Development and Training Core (CDTC) will serve as a local and regional scientific career development hub that aims to create a pipeline of diverse scholars who are committed to community engagement and expertise in interdisciplinary, multi-sector research partnerships. The CDTC aims to create a hub for mental health training opportunities and resources in structural racism, cultural humility, racial allyship, and diversity, equity, inclusion, and justice. ESI scholars will be engaged in a hands-on training that has a focus on being community-led, anti-racism and interdisciplinary. Strengths include that the CDTC has clear plans of focus on early stage and under-represented and minoritized investigators and focus on preparing scholars for health systems change and solutions-oriented research to impact policy. There is an explicit focus on training in anti-racism and cultural and structural training required to foster sustainable innovation and hands on-intervention science experiences. NY-CHAMP has a clear training philosophy that values co-creation and co-implementation alongside the diverse communities being served by NY-CHAMP, and this is infused throughout the proposal but essentially in the CDTC plans. The leadership has a strong track record in supporting career development programs focused on maternal health equity. Combining ESIs, senior leadership, and community investigators throughout projects is innovative. ESIs and new investigators in leadership roles may need additional support and protected time across projects. Given already extensive mentoring roles of leadership, a clear plan for protected time for mentorship of senior faculty is needed. Strong institutional support with Columbia and Cornell committing two $20k pilot awards per year in Years 2-5 of the grant for ESIs. Visiting scholars' program that includes community partners is a strength.

Research Project 1

Principal Investigator(s): Pathak, Jyotishman

Criteria Scores:

Significance: 1
Investigator: 1
Innovation: 1
Approach: 3
Environment: 1

Overall Impact: Project 1 proposes to integrate clinical data with SDoH data derived from the Patient-Centered Outcomes Research Institute (PCORI)-funded INSIGHT Clinical Research Network (Project Lead Pathak) on >328,000 women across New York City (NYC). The proposal will first link EHRs and claims data then link population-level SDoH data for the study cohort using natural language processing and machine learning. This data will be used to predict risk of insufficient prenatal care and SMM, and assessments of bias using machine learning. Then the team will assess the usability and acceptability of a pilot integration of risk models into point-of-care clinical decision support (CDS) at two study sites (Weill Cornell Medicine and Columbia University Irving Medical Center) using patient and community input to inform development and use. Score-driving strengths include innovative methods (machine learning, natural language processing) integrated with both SDoH, claims, and clinical record data using a multi-level perspective that also accounts for clinician bias; an excellent investigator team with the requisite experience to conduct this trial, and a clear plan for how study findings will translate into clinical applications. Minor score-driving weaknesses in the approach include some concerns regarding timeline for Aim 4 clinical implementation if there are delays in Aims 1-3 leading to the development of the tool, and some limitations noted in using clinical records to derive SDoH data. These weaknesses do not detract from the high potential impact of this research project.

1. Significance:

Strengths

- Addresses key gaps in prior work by integrating SDoH data and existing clinical record data to predict who may be most likely to experience inadequate prenatal care and have complications using multilevel perspective.
- Risk assessments rarely account for both individual- and population-level measures.
- Proposal to translate findings from Aims 1-3 into a CDS tool with plans for community input on the adaptation and buy-in.

Weaknesses

- None noted.

2. Investigator(s):

Strengths

- Expertise in the required methods and access to relevant data sources.
- PI Dr. Pathak is an expert in mental health informatics and has extensive experience with data mining using EHRs and claims data.
- Dr. Xiao, an early-stage investigator, has expertise in linking multilevel and longitudinal data to study social determinants of mental health among health disparity populations.
- Co-I's (Benda) has expertise in human-centered design and developing CDS with a maternal health equity framework.
- Project Lead Freidman at Columbia has grants evaluating HER risk prediction of adverse inpatient obstetrical events.
- Sevonna Brown from Black Women's Blueprint will serve as a Community Collaborator on this project and bring expertise in racial healing, interventions for health system and provider bias, and policy codesign for maternal health equit.

Weaknesses

- None noted.

3. Innovation:

Strengths

- Applies machine learning to develop multi-level risk models for predicting inadequate prenatal care and integrates electronic health records (EHRs), health insurance claims, and community/neighborhood- and individual-level SDoH data.
- Includes individual-level SDoH data derived from clinical narratives in the EHRs using machine learning/natural language processing approaches.
- Proposes a clinical decision support tool to translate findings from Aims 1-3 to clinical practice that leverages machine learning innovations and community input.

Weaknesses

- None noted.

4. Approach:

Strengths

- Incorporates longitudinal EHR and claims data from a large, diverse population (n of >328,000 patients).

- Integrates individual-level SDoH data derived from clinical narratives in the EHRs using machine learning/natural language processing approaches with these data sources.
- Engages community partners in developing and implementing the fairness approaches employed in the machine learning models.
- Also engages community partners in feedback on piloting the risk tool (Aim 4).
- Builds upon strong multi-level conceptual framework.

Weaknesses

- Minor: Definition of "inadequate" prenatal care may not be patient-centered.
- Unclear how findings from all three first Aims will be translated into the CDS tool in Aim 4 and how it could impact timeline.
- Clinical notes may be limited quality and/or limited information about planned SDoH factors.

5. Environment:

Strengths

- Strong institutional data core at Cornell to support the proposed aims.

Weaknesses

- None noted.

Study Timeline:

Strengths

- None noted.

Weaknesses

- Unclear how findings from all three first Aims will be translated into the CDS tool in Aim 4 and how it could impact timeline.

Protections for Human Subjects:

Acceptable Risks and Adequate Protections

Data and Safety Monitoring Plan (Applicable for Clinical Trials Only):

Acceptable

Inclusion Plans:

- Sex/Gender: Distribution justified scientifically
- Race/Ethnicity: Distribution justified scientifically
- For NIH-Defined Phase III trials, Plans for valid design and analysis: Not applicable
- Inclusion/Exclusion Based on Age: Distribution justified scientifically

Vertebrate Animals:

Not Applicable (No Vertebrate Animals)

Biohazards:

Not Applicable (No Biohazards)

Resource Sharing Plans:
Acceptable

Authentication of Key Biological and/or Chemical Resources:
Not Applicable (No Relevant Resources)

Budget and Period of Support:
Recommend as Requested

Research Project 2
Principal Investigator(s): Osborne, Lauren

Criteria Scores:
Significance: 1
Investigator: 2
Innovation: 2
Approach: 4
Environment: 1

Overall Impact: Project 2 is a Hybrid Type 1 Effectiveness-Implementation Trial evaluating the Living Healthy for Moms doula-led intervention. The two-part intervention focuses first on care continuity and community-to-healthcare linkages during the week following discharge as a critical window. The second part is a package of evidence-based interventions to address community priorities, including an evidence-based online empowerment program focused on building patient self-advocacy, a doula led health emergency detection program, and a 12-session trauma informed doula phone intervention that integrates CBT for depression and skills to detect risk and linkage to care for additional CV support. Score-driving strengths include innovations around the use of the doula workforce to extend access to evidence-based care using an integrated behavioral health care model, strong investigator team and excellent environment. Score-driving weaknesses in the approach include lack of clarity regarding the formative work necessary to translate the CBT program for doula delivery; lack of ability to disentangle which intervention components may be driving any effects detected; under-developed implementation science outcomes; and more information needed on the selection of and supervision plans for doulas and how to balance confidentiality in close knit communities as doulas were selected from the communities being served. Overall, these moderate weaknesses in the Approach do not detract from the high potential impact of this project.

1. Significance:
Strengths
- Doulas are a workforce that may extend access to evidence-based care but are underutilized and evaluations of evidence-based doula-interventions to date have been limited.

Weaknesses

- Given the range of intervention components to be evaluated, it is possible that the design will not be able to disentangle which component is driving any effects.

2. Investigator(s):

Strengths

- Team brings expertise in maternal MH (Osborne, Monk), CBT (Osborne, Monk), obstetrics/maternal–fetal medicine (Reddy, Lipkind, Nathan, Booker Goffman, Aubey), CVD (Safford, Lipkind, Miller, Booker,), public health (Hall, Safford, Reddy), community engagement/ community health worker programs (Safford, Cepin, Hall, Dorvall, St. Clair), health behavioral theory (Safford, Hall), clinical trials (Reddy, Lipkind, Safford, Osborne), implementation science and mixed methods (Hall, Shelton, Safford, Christos), data analysis (Christos, Osborne, Safford, Reddy), and doula care (Cepin, Dorvall, St. Clair).
- The team shares strong histories of NIH funding; leadership of large-scale research projects, programs, and centers; national reputations for relevant scientific and clinical contributions; successful track records of collaboration with one another and with large teams; and strong institutional environments and resources.

Weaknesses

- None noted.

3. Innovation:

Strengths

- Doulas are under-utilized yet desire new skills and there is an opportunity to extend evidence-based interventions using doula delivery.
- Embedding a mental health component in a CV health behavior change intervention offers a novel approach to overcome the stigma associated with MH that is highly prevalent in the targeted population.

Weaknesses

- Given the range of intervention components evaluated in P2, it is possible design will not be able to disentangle which component is driving any effects.
- Lack of clarity provided on how adaptations of CBT components for doula delivery will promote innovation.

4. Approach:

Strengths

- Leverage doula workforce using a multilevel intervention approach.
- Designed with community input and involvement from stakeholder partners, propose iterative plan for intervention adaptation.

Weaknesses

- Unclear how extensive the formative work is that is needed to further adapt the intervention and integrate components, including lack of attention to specific adaptations to the Thinking Healthy CBT program for doula delivery that will be necessary. For instance, considerations around language, translation, and accessibility of cognitive concepts. Also unclear the extent to which formative work will involve tailoring adaptations to each of the three sites.

- Lack of attention to the modality of delivery (in person vs. virtual) and which is most acceptable to the most at-risk high need patients who may not have adequate privacy for virtual options or considerations of uptake of an online program in the immediate postpartum period.
- Some implementation science design considerations underdeveloped, including how implementation outcomes are defined and evaluated -- implementation science outcomes limited (e.g., relying only client satisfaction questionnaire) – measures not guided by the stated frameworks of CFIR and RE-AIM.
- Description of attention control condition is underdeveloped.
- 47% lower incidence of PP depression compared to attention control may be overstated for this population and power estimates also do not include the extensive number of analyses proposed.
- Qualitative and quantitative data collection is proposed without a clear plan for integrating qual and quant data.
- Design does not allow for disentangling which components are driving intervention effects and intervention has diffuse focus of outcomes related to PPD, CV, well-being, health care utilization and satisfaction.
- More information is needed on the selection of doulas and how to balance confidentiality in close knit communities as doulas were selected from the communities being served.
- The intervention and study utilize several conceptual models without clear plans for integrating these models.
- Supervision plan for doulas underdeveloped. Additionally, contamination risk depending on level of communication and collaboration across doulas.

5. Environment:

Strengths

- Columbia and Cornell have integrated mental health care platforms within OB/GYN settings that offer a strong setting to do this work.

Weaknesses

- Unclear how findings will generalize to other contexts.

Study Timeline:

Strengths

- Doulas have already been selected suggesting feasibility of startup.

Weaknesses

- One year may not offer sufficient time to refine the intervention.

Protections for Human Subjects:

Acceptable Risks and Adequate Protections

Data and Safety Monitoring Plan (Applicable for Clinical Trials Only):

Acceptable

Inclusion Plans:

- Sex/Gender: Distribution justified scientifically
- Race/Ethnicity: Distribution justified scientifically
- For NIH-Defined Phase III trials, Plans for valid design and analysis: Not applicable
- Inclusion/Exclusion Based on Age: Distribution justified scientifically

Vertebrate Animals:
Not Applicable (No Vertebrate Animals)

Biohazards:
Not Applicable (No Biohazards)

Resource Sharing Plans:
Acceptable

Authentication of Key Biological and/or Chemical Resources:
Not Applicable (No Relevant Resources)

Budget and Period of Support:
Recommend as Requested

Research Project 3
Principal Investigator(s): Li, Guohua

Criteria Scores:
Significance: 1
Investigator: 1
Innovation: 2
Approach: 4
Environment: 1

Overall Impact: Few studies have empirically evaluated the role of structural racism in morbidity and mortality among racial/ethnic minority individuals nor evaluated the impact of specific policy interventions at multiple levels. Project 3 proposes mixed methods to 1) assess the associations of multidimensional indexes of SR with racial and ethnic disparities in SMM, suicidality, and mortality; and estimate the hospital costs of SMM attributable to SR; 2) assess the associations of two policy-relevant interventions (i.e., Medicaid expansion and healthcare workforce diversity) with racial and ethnic disparities in SMM, suicidality, and mortality; and quantify the moderating effects of these two interventions on associations between SR and maternal health outcomes; and 3) analyze the lived experiences of patients and community and hospital stakeholders to identify the mechanisms of, and effective solutions for, SR in maternal health care. Data from the Healthcare Cost and Utilization Project, which provides information on 10 million childbirths from over 1200 hospitals in 21 states

during 2016-2022 (Aims 1-2), will be complemented by prospective in-depth interview and focus group data from multisector stakeholders (patients, community, hospital). This proposal is led by an excellent team of investigators with relevant prior work, the necessary expertise to conduct this research. Score-driving weaknesses in the approach related to limited measurement of SR and the lack of mixed methods analysis proposed to integrate qualitative and quantitative data. Despite these limitations, the project is likely to have moderate impact on the field.

1. Significance:

Strengths

- Findings will help close important gaps in understanding the role that SR plays in SMM/MM disparities and generate urgently needed evidence for informing multilevel policy interventions to improve maternal health equity.
- More research is needed on policy solutions to address impact of structural racism on racial/ethnic minority maternal health outcomes.
- Contributes critical, timely science to generate new evidence that can transform the systems and policy solutions driving U.S. maternal health inequities.
- Establishing a research database for 10 million childbirths in 21 states is a major data resource contribution.

Weaknesses

- None noted.

2. Investigator(s):

Strengths

- Excellent multidisciplinary team of investigators with the relevant expertise to conduct this study. PI Li's long track record of NIH research focuses on population-based and policy-oriented studies that encompass novel epidemiologic designs, innovative analytical approaches, and complex data systems.
- Dr. Hall (NY-CHAMP Overall MPI) has expertise in social and structural determinants of maternal health disparities, and policy and health systems interventions. She has expertise in community engagement, policy evaluation, and quantitative and mixed methods.
- Co-I team includes relevant expertise: maternal fetal medicine and electronic health record analyses (Dr. Friedman); health policy, econometrics and Medicaid (Dr. Daw); discrimination, xenophobia and racism and public health (Dr. Samari); health economics, cost-effectiveness, and mental health (Dr. Jalali); biostatistics (Dr. Chen); and the government, American politics, Medicaid, and racial disparities (Dr. Michener). Strong history of collaboration across team members.
- Team was among the first to link state-level healthcare workforce diversity to reduced risk of severe adverse maternal outcomes.
- NY-CHAMP Community MPI Sevonna Brown from Black Women's Blueprint will serve as a Community Collaborator on this project.

Weaknesses

- Minor: unclear how Dr. Guglielminotti's pending R01, Structural Racism and Maternal Health Disparities overlaps with this proposal.
- Community MPI Sevonna Brown is also lead community PI in Project 1 without clear plans for ensuring Brown has sufficient time for both projects, as well as engaging other community MPIs.

3. Innovation:

Strengths

- Integrating qualitative data collection with a robust quantitative data set from the Healthcare Cost and Utilization Project, which provides information on 10 million childbirths from over 1200 hospitals in 21 states is innovative. National quantitative data interpreted alongside findings from qualitative data, in partnership with local community and health care partners, provides a holistic view of how system factors affect maternal health care outcomes.
- Examines timely, actionable policy interventions for mitigating the impact of structural racism on maternal health disparities (i.e., expanding Medicaid and healthcare workforce diversification).
- Provides innovative data on systems-level cost analyses of policy interventions to address structural racism.

Weaknesses

- Approach to measuring structural racism is not novel .

4. Approach:

Strengths

- Preliminary studies on state- and hospital-level indicators of SR and SMM as well as qualitative work from a needs assessment.
- Proposal builds off of a well-articulated conceptual framework that also includes cost outcomes and hospital indicators.
- Large dataset from the Healthcare Cost and Utilization Project, which provides information on 10 million childbirths from over 1200 hospitals in 21 states is a great resource to provide sufficient statistical power to assess policy impact on maternal mortality.

Weaknesses

- Plans to integrate qualitative and quantitative using mixed methods analysis are not clearly specified.
- Structural racism is measured using overlapping constructs and is limited by the data sources proposed.

5. Environment:

Strengths

- Excellent environment with the resources necessary to conduct this study.

Weaknesses

- None noted.

Study Timeline:

Strengths

- None noted.

Weaknesses

- Timeline for final aim may be delayed depending on progress of early aims.

Protections for Human Subjects:

Acceptable Risks and Adequate Protections

Inclusion Plans:

- Sex/Gender: Distribution justified scientifically
- Race/Ethnicity: Distribution justified scientifically
- For NIH-Defined Phase III trials, Plans for valid design and analysis: Not applicable
- Inclusion/Exclusion Based on Age: Distribution justified scientifically

Vertebrate Animals:
Not Applicable (No Vertebrate Animals)

Biohazards:
Not Applicable (No Biohazards)

Resource Sharing Plans:
Not Applicable (No Resource Sharing Plans)

Authentication of Key Biological and/or Chemical Resources:
Not Applicable (No Relevant Resources)

Budget and Period of Support:
Recommend as Requested

CRITIQUE 2:

Significance: 1
Investigator(s): 1
Innovation: 2
Approach: 3
Environment: 1
Community Partnership Component: 2
Training Component: 2
Research Project 1: 4
Research Project 2: 2
Research Project 3: 3

Overall Impact: The New York (NY) Community–Hospital–Academic Maternal Health Equity Partnerships (NY-CHAMP) Maternal Health Research Center of Excellence aims to change the maternal health care ecosystem in New York City and the broader state region. While this is a lofty aim,

the team's leadership and environment are exceptional and the proposal includes many prominent community organizations, initiatives, etc. The MPIs include nine total individuals, half of whom are academics (nearly all of whom are Fully Professors) and half of whom are community leaders. The size of the leadership team could be a challenge and some of the MPIs have limited experience leading a similar-sized project; additionally, it was unclear how the leadership team would all coordinate together. The research projects are strong and distinct but also not entirely complimentary. The research projects focus on cardiovascular health and mental health. Finally, there are solid plans to engage early career colleagues through the training component. Score driving strengths included the exceptional leadership and environment as well as the research projects along with the significance. Score driving weaknesses included some limitations within the research projects and the large MPI team. Overall, the potential impact is high.

1. Significance:

Strengths

- Focal point on cardiovascular and mental health is highly significant.
- The locality of New York City and New York State is appropriate to address the striking maternal health disparities, particularly affecting Black women.
- The reach of the team, including within the community, raises the significance as solutions could be implemented throughout many levels.

Weaknesses

- None noted.

2. Investigator(s):

Strengths

- The MPI team includes nine individuals, half of whom are academics (split between Columbia and Cornell) and half of whom are community leaders; each has a role in one of the other reseach projects and/or components.
- MPIs have complementary expertise (e.g., public health, maternal-fetal medicine) and many bring lived experience.
- A number of MPIs have already collaborated.
- Plan to have a Steering Committee, a community board (i.e., CHARGE Board), central. staff/administrators, External Scientific Advisory Board.
- Appears the Center and project aims were planned with academic and community partners.

Weaknesses

- The size of the MPI team is quite large and not entirely justified, no plans to coordinate in person.
- While there are a number of Full Professors with extensive experience leading similarly scaled projects, some people are early career and/or new to the organization such as MPI St. Claire; the academic and community MPI model is a sign that the team is committed to being equitable and yet, 9 people will make that difficult and there are few plans to support the early-career MPIs.
- Contact MPI Reddy has extensive experience and is well-positioned to lead this project, however she joined Columbia a year ago so her community ties may be limited; a few other MPIs are also relatively new to their organizations; Reddy may be stretched too thin given other commitments so it would be helpful to see more clear plans to support other MPIs to lead.

3. Innovation:

Strengths

- Integrate various methodological frameworks (e.g., weathering hypothesis, social ecological).
- Ground their community and training components in anti-racism and other relevant frameworks.
- The population is heavily burdened.
- Include rural and urban populations.
- Focus on structural issues, using mixed methods.
- Various research projects have innovative aspects (e.g., using AI to predict patient risk).

Weaknesses

- None noted.

4. Approach:

Strengths

- Approach varies across the projects but are generally strong such as those related to implementation science and AI.
- Incorporate lived experiences.
- Builds off of the existing Maternal Hospital Quality Improvement Network.

Weaknesses

- The research projects would be linked together in better ways, for example, it is not clear how the findings from one might inform the others.
- Sustainability of some of the interventions is not addressed.

5. Environment:

Strengths

- Columbia and Cornell have both committed $160k in pilot awards for this work.
- Also includes four well-established community organizations.

Weaknesses

- While the organizations are all within New York City, they are spread across boroughs so some plan to connect in person on a semi-regular basis would have strengthened the proposal.

Study Timeline:

Strengths

- Appropriate.

Weaknesses

- None noted.

Protections for Human Subjects:

Acceptable Risks and Adequate Protections

Inclusion Plans:

- Sex/Gender: Distribution justified scientifically

- Race/Ethnicity: Distribution justified scientifically
- For NIH-Defined Phase III trials, Plans for valid design and analysis: Not applicable
- Inclusion/Exclusion Based on Age: Distribution justified scientifically

Vertebrate Animals:
Not Applicable (No Vertebrate Animals)

Biohazards:
Not Applicable (No Biohazards)

Resource Sharing Plans:
Acceptable

Authentication of Key Biological and/or Chemical Resources:
Acceptable

Budget and Period of Support:
Recommend as Requested

Community Partnership Component
Principal Investigator(s): Hall, Cepin

Impact:
- Led by Hall (Associate Professor of Public Health) and Cepin (Associate Professor of Obstetrics and Gyn), have 5 other Co-Is.
- Centers community members, some of whom are in leadership roles, so it appears as there is truly a cooperative and equitable partnership.
- Plan to develop innovative community-led maternal health provider + birthing patient training program.
- At one of the community partners is a community healthcare partner.
- Co-designing the deliverables should ensure that communities are engaged.

Training Component
Principal Investigator(s): Taylor, Monk

Impact:
- Have proposed they will become a career development hub, particularly with the goal to create a pipeline of diverse ESIs; will train ESIs to be interdisciplinary and work *with* communities.

- Will pair mentors and mentees but few details of evidence-based mentor training curriculum (for mentee or mentor).
- Leads have extensive experience with mentoring.
- Columbia and Cornell have committed to $20k in years 2-5 for ESIs.

Research Project 1

Principal Investigator(s): Pathak, Xiao

Criteria Scores:

Significance: 1
Investigator: 1
Innovation: 1
Approach:4
Environment: 1

Overall Impact: This is a highly significant study given the focus on integrating clinical data with SDoH data derived from the Patient-Centered Outcomes Research Institute (PCORI)-funded INSIGHT Clinical Research Network. This would be done among almost 400,000 women across New York City by leveraging machine learning and natural language processing techniques. The combination of EHR, claims, and SDoH data to develop these novel AI risk models will then be used predict severe maternal morbidity and mortality associated with cardiovascular and mental health outcomes. Score-driving strengths included the significance, investigators, and environment. Score-driving weaknesses included limitations in the approach. Overall, the potential impact is high.

1. Significance:

Strengths

- Gaps in prior work where SDoH could not be examined alongside clinical data will be addressed.
- Will elucidate individual-level and population-level factors.

Weaknesses

- None noted.

2. Investigator(s):

Strengths

- Well-qualified team with complementary expertise, including experts in mental health informatics (Project Lead Pathak); other Project Lead is an ESI (Xiao) who will help link multilevel and longitudinal data.
- Co-Is includes along with those with expertise in designing clinical decision support (CDS) tools.
- Community partner with Co-I Brown from Black Women's Blueprint brings expertise in health system interventions and other relevant areas.

Weaknesses

- None noted.

3. Innovation:

Strengths

- Data are robust.
- CDS has not been used much to understand maternal health.

Weaknesses

- None noted.

4. Approach:

Strengths

- Application of machine learning techniques all seem appropriate.
- Community partners will help address fairness within the machine learning models and a number of other steps.
- Solid conceptual framework.

Weaknesses

- Did not thoroughly address the impact of low-quality data in EHRs (e.g., few notes in a social history about SDoH).
- The CDS tool in aim 4 may not be well-informed by the prior aims.

5. Environment:

Strengths

- Strong environment.

Weaknesses

- None noted.

Study Timeline:

Strengths

- Appropriate

Weaknesses

- None noted.

Protections for Human Subjects:

Acceptable Risks and Adequate Protections

Inclusion Plans:

- Sex/Gender: Distribution justified scientifically
- Race/Ethnicity: Distribution justified scientifically
- For NIH-Defined Phase III trials, Plans for valid design and analysis: Not applicable
- Inclusion/Exclusion Based on Age: Distribution justified scientifically

Vertebrate Animals:

Not Applicable (No Vertebrate Animals)

Biohazards:
Acceptable

Resource Sharing Plans:
Acceptable

Authentication of Key Biological and/or Chemical Resources:
Not Applicable (No Relevant Resources)

Budget and Period of Support:
Recommend as Requested

Research Project 2
Principal Investigator(s): Osborne, Reddy, Safford, Lipkind

Criteria Scores:
Significance: 1
Investigator: 1
Innovation: 1
Approach: 2
Environment: 1

Overall Impact: This is a highly significant study given the focus on using doulas to deliver care during the critical 7-day postpartum day window and into the first six months postpartum. The intervention would not only include a focus on CVD outcomes but also mental health and would leverage doulas from within the community. Score-driving strengths included the significance, investigators, and environment. Score-driving weaknesses included limitations in the approach, such as being unable to distinguish the different effective aspects of the intervention from one another. Overall, the potential impact is high.

1. Significance:

Strengths

- Preventing CVD and mental health disparities are critical to improving maternal health.
- If the intervention is effective, could change care approach.

Weaknesses

- None noted.

2. Investigator(s):

Strengths

- Exceptional team with the necessary expertise including in obstetrics, maternal health, doula care, CVD, mental health (especially cognitive behavioral therapy), public health, community engagement, clinical trials, implementation science.
- History of collaboration and long-NIH funding track records.

Weaknesses

- None noted.

3. Innovation:

Strengths

- Doula care can fit a critical gap during this vulnerable period and it’s understudied and underutilized.
- Weaving in mental health care within a CVD intervention is novel.
- Examining multiple outcomes.
- Getting community input.

Weaknesses

- None noted.

4. Approach:

Strengths

- Delivering care via doulas.
- Community input will shape the intervention.

Weaknesses

- With the different interventions, unclear how will pinpoint which element(s) are most important.
- Unclear how much the Thinking Healthy CBT program will need to be adapted for this intervention.
- The statistical power calculations seemed they may have been overestimated.

5. Environment:

Strengths

- Strong environment.

Weaknesses

- None noted.

Study Timeline:

Strengths

- Appropriate.

Weaknesses

- None noted.

Protections for Human Subjects:

Acceptable Risks and Adequate Protections

Data and Safety Monitoring Plan (Applicable for Clinical Trials Only):
Acceptable

Inclusion Plans:

- Sex/Gender: Distribution justified scientifically
- Race/Ethnicity: Distribution justified scientifically
- For NIH-Defined Phase III trials, Plans for valid design and analysis: Not applicable
- Inclusion/Exclusion Based on Age: Distribution justified scientifically

Vertebrate Animals:
Not Applicable (No Vertebrate Animals)

Biohazards:
Not Applicable (No Biohazards)

Resource Sharing Plans:
Acceptable

Authentication of Key Biological and/or Chemical Resources:
Not Applicable (No Relevant Resources)

Budget and Period of Support:
Recommend as Requested

Research Project 3
Principal Investigator(s): Li, Hall, Guglielminotti

Criteria Scores:
Significance: 1
Investigator: 1
Innovation: 1
Approach: 4
Environment: 1

Overall Impact: This is a highly significant study given the focus on how structural racism shapes maternal health. The study draws on data across the country with quantitative data from 10 million births as well as qualitative data in New York. Score-driving strengths included the significance, investigators, and environment. Score-driving weaknesses included limitations in the approach such as

not tying together the quantitative and qualitative data from the different geographic locations. Overall, the potential impact is high.

1. Significance:

Strengths

- Will elucidate social and structural determinants of maternal health, particularly focused on structural racism.
- We know little about the policy solutions, let alone the financial cost of structural racism.
- Findings can inform multilevel interventions.

Weaknesses

- None noted.

2. Investigator(s):

Strengths

- Exceptional team with expertise in various fields (epidemiology, maternal health, obstetrics, health services research, health policy, econometrics, discrimination, disparities).
- Many members with experience leading large-scale NIH grants, includes some early-career investigators.
- Strong collaboration history.
- Has community collaborator.

Weaknesses

- None noted.

3. Innovation:

Strengths

- Combining qualitative and quantitative data is innovative on this topic particularly with the size of the quant data (e.g., 10 million births).
- Can examine multi-level systems.
- Can inform immediate actions like Medicaid expansion.

Weaknesses

- None noted.

4. Approach:

Strengths

- Compelling preliminary data.
- Informed by conceptual framework.
- Mix of the large quantitative dataset with the more local qualitative.

Weaknesses

- Unclear how the national quantitative data will tie into the qualitative data that is only local.
- Concern about the measures of structural racism being blunt (e.g., at the county and state-level rather than the census-track or something more granular).
- Many of the measures of structural stigma seem they will be co-linear with one another and do not truly capture the underlying construct.

5. Environment:

Strengths

- Strong environment.

Weaknesses

- None noted.

Study Timeline:

Strengths

- Appropriate

Weaknesses

- None noted.

Protections for Human Subjects:

Acceptable Risks and Adequate Protections

Inclusion Plans:

- Sex/Gender: Distribution justified scientifically
- Race/Ethnicity: Distribution justified scientifically
- For NIH-Defined Phase III trials, Plans for valid design and analysis: Not applicable
- Inclusion/Exclusion Based on Age: Distribution justified scientifically

Vertebrate Animals:

Not Applicable (No Vertebrate Animals)

Biohazards:

Not Applicable (No Biohazards)

Resource Sharing Plans:

Acceptable

Authentication of Key Biological and/or Chemical Resources:

Acceptable

Budget and Period of Support:

Recommend as Requested

CRITIQUE 3:

Significance: 2
Investigator(s): 1
Innovation: 2
Approach: 2
Environment: 1
Community Partnership Component: 1
Training Component: 1
Research Project 1: 5
Research Project 2: 2
Research Project 3: 1

Overall Impact: This center proposal includes one-of-a-kind collaborative that is inclusive a range of community-based organizations, academic medical centers, and hospital systems across the entire state of New York. The team has engagements with almost every initiative related to maternal M&M in NYS. Grounded in Research Justice theories, the center has a genuine commitment to maternal health equity and has a high potential impact positive change in the community. Two out of the three research projects are exceptionally strong and well-designed.

1. Significance:

Strengths

- The overall center proposal is strongly focused on preventable health issues (cardiovascular and mental health conditions) that are attributable to disparities in maternal health including severe maternal morbidities. Their preliminary data implicating structural racism and maternal inequities are compelling and provides a promising ground for generating highly significant empirical evidence.
- The proposal demonstrates a strong, tight-knit, collaborative between hospital systems, community partners with assets in key maternal care providers such as doulas and nurse midwives, and academic medical centers. Such an initiative has potential to develop long-lasting and impactful community relationships and truly advance maternal health care practices.

Weaknesses

- While the proposal emphasizes addressing community-level inequities and seeks to reform systems and policy related to maternal care, the overall project seems to have overlooked key safety and policy issues related to abortion care. Despite each of the following strengths – (1) MPI Hall's work on association between abortion and mental health, (2) a dedicated project on policy issues and policy reform (project 3), (3) mental health as one of the overarching themes of the proposal, and (4) inclusion of and partnerships with community-based women's health providers – despite all of these opportunities and strengths, the proposal (and research project 3) may have missed an opportunity to address a pressing maternal care issue in the region as a part of this proposal.

2. Investigator(s):

Strengths

- The 9-member MPI team are highly qualified with complementary and deep expertise in various domains (maternal-fetal medicine, implementation science, health equity, community engagement, etc.), are all-women (based on pronouns used in narrative), racially-ethnically diverse (based on background description), and come from different regional institutions in NYS, all of which are critical for a center of excellence in maternal health.

Weaknesses

- One MPI (St. Clair) is a recent BA graduate without much leadership experience. They are a program director at one of the community partner organizations, a role they recently assumed, as well as a graduate student in public health. An MPI role is not well-justified from research and experience standpoints. However, they are an experienced doula, which may be one of the reasons they were included in an MPI capacity.

3. Innovation:

Strengths

- Moving away from traditional hospital-based interventions and focusing on SDoH factors and structural issues such as racism is inherently innovative.

Weaknesses

- None noted.

4. Approach:

Strengths

- The overall approach of co-designing for equity is thoughtful and much needed for a project of this scale.

Weaknesses

- There are methodological issues with research project 1, which are documented separately.

5. Environment:

Strengths

- The project has rich and extensive resources and collaborations needed for the development and sustainability of the center.

Weaknesses

- None noted.

Study Timeline:

Strengths

- None noted.

Weaknesses

- None noted.

Protections for Human Subjects:

Acceptable Risks and Adequate Protections

Inclusion Plans:

- Sex/Gender: Distribution justified scientifically
- Race/Ethnicity: Distribution justified scientifically
- For NIH-Defined Phase III trials, Plans for valid design and analysis: Not applicable.
- Inclusion/Exclusion Based on Age: Distribution justified scientifically

Vertebrate Animals:
Not Applicable (No Vertebrate Animals)

Biohazards:
Not Applicable (No Biohazards)

Resource Sharing Plans:
Acceptable

Authentication of Key Biological and/or Chemical Resources:
Not Applicable (No Relevant Resources)

Budget and Period of Support:
Recommend as Requested

Community Partnership Component
Principal Investigator(s): Reddy, Uma

Impact: The community partnership component is the strongest element of this element as described in other sections.

Training Component
Principal Investigator(s):

Impact: The project has an effective and culturally relevant training plan and includes early-stage investigators as one of the major participants.

Research Project 1
Principal Investigator(s):

Criteria Scores:

Significance: 2
Investigator: 1
Innovation: 5
Approach: 5
Environment: 1

Overall Impact: Project 1 is technically sound with moderate concerns to methodological issues related to bias mitigation and interpretability and, therefore, has moderate to low impact as compared to other research projects.

1. Significance:

Strengths

- The prediction outcomes are quite comprehensive and inclusive of severe maternal morbidities and prenatal adequacy.

Weaknesses

- None noted.

2. Investigator(s):

Strengths

- The investigators and project leads have the necessary background and expertise in health informatics, maternal care and adverse outcomes, and predictive modeling.

Weaknesses

- None noted.

3. Innovation:

Strengths

- None noted.

Weaknesses

- The proposed methods and resources are not necessarily innovative per se. While some aspects such as using a common data model and phenotyping methods are appropriate, there is little to no emphasis on privacy and data linkage issues. Linking clinical, claims, and SDoH are highly risky and true de-identification is not possible. Such linking also has potential to amplify and perpetuate inequities and may work against the overall values and objectives of the Center.

4. Approach:

Strengths

- The overall approach and methods, except for interpretability and bias mitigation aspects, are informatics-savvy i.e., it has all the pieces needed for an ML-based project (CDM, phenotyping, FHIR-based tool, etc.).

Weaknesses

- The proposed self-supervised models are inherently not interpretable and expensive to translate into a clinical decision support tool without broad application. Using methods such as SHAP may help, but not in ways as proposed in the application, where SHAP is being utilized for bias detection.
- Instruments such as SUS are not designed to capture the nuances and complexities of ML models and FHIR apps.

5. Environment:

Strengths

- The project has access to necessary resources (e.g., clinical data warehouse, INSIGHT) and informatics infrastructure.

Weaknesses

- None noted.

Study Timeline:

Strengths

- None noted.

Weaknesses

- None noted.

Protections for Human Subjects:

Unacceptable Risks and/or Inadequate Protections

- Data linkage and reidentification risks are not addressed sufficiently

Inclusion Plans:

- Sex/Gender: Distribution justified scientifically
- Race/Ethnicity: Distribution justified scientifically
- For NIH-Defined Phase III trials, Plans for valid design and analysis: Not applicable
- Inclusion/Exclusion Based on Age: Distribution justified scientifically

Vertebrate Animals:

Not Applicable (No Vertebrate Animals)

Biohazards:

Not Applicable (No Biohazards)

Resource Sharing Plans:

Acceptable

Authentication of Key Biological and/or Chemical Resources:
Not Applicable (No Relevant Resources)

Budget and Period of Support:
Recommend as Requested

Research Project 2
Principal Investigator(s): Reddy, Uma

Criteria Scores:
Significance: 1
Investigator: 1
Innovation: 1
Approach: 2
Environment: 1

Overall Impact: Project 2 (randomized trial of doula-led postpartum interventions) is an exemplar of the kinds of research projects that a center of “excellence” in maternal health would nurture and lead. Through authentic collaborations with community-based organizations who are committed to healthy equity and a rigorous intervention trial, this project focuses on generating evidence related to doula-led post-partum interventions and has high potential to impact positive change in maternal health outcomes.

1. Significance:
Strengths
- Mental health and cardiovascular issues are highly prevalent among postpartum women, yet only around 6% receive sufficient and appropriate treatment. This project has the potential to generate actionable evidence for implementing out-of-hospital care post-partum.

Weaknesses
- None noted.

2. Investigator(s):
Strengths
- This project has an exceptional, multidisciplinary team of investigators with complementary expertise in maternal-fetal medicine, cognitive behavioral therapy, and implementation science to successfully design and execute the proposed intervention trial.

Weaknesses
- None noted.

3. Innovation:
Strengths

- The proposed intervention is multi-tiered and focused specifically on the first 7 days post discharge. A participatory-based research approach to test the intervention and a comprehensive set of outcome measures has the potential to shift the focus from basic support services and handoffs to robust care continuity for birthing individuals and their families.

Weaknesses

- None noted.

4. Approach:

Strengths

- The intervention trial is thoughtful designed from recruitment, implementation to rigorous outcome measurement and analyses plan.

Weaknesses

- None noted.

5. Environment:

Strengths

- The project has the necessary partnerships and resources needed to execute the project.

Weaknesses

- None noted.

Study Timeline:

Strengths

- None noted.

Weaknesses

- None noted.

Protections for Human Subjects:

Acceptable Risks and Adequate Protections

Inclusion Plans:

- Sex/Gender: Distribution justified scientifically
- Race/Ethnicity: Distribution justified scientifically
- For NIH-Defined Phase III trials, Plans for valid design and analysis: Not applicable
- Inclusion/Exclusion Based on Age: Distribution justified scientifically

Vertebrate Animals:

Not Applicable (No Vertebrate Animals)

Biohazards:

Not Applicable (No Biohazards)

Resource Sharing Plans:
Acceptable

Authentication of Key Biological and/or Chemical Resources:
Not Applicable (No Relevant Resources)

Budget and Period of Support:
Recommend as Requested

Research Project 3
Principal Investigator(s): Reddy, Uma

Criteria Scores:
Significance: 1
Investigator: 1
Innovation: 1
Approach: 1
Environment: 1

Overall Impact: Project 3 seeks to study and address important structural issues related to maternal health. Except for one moderate concern related to exclusion (or lack of mention) of clinically relevant abortion care and related policy issues, the project is well-designed with appropriate resources.

1. Significance:

Strengths

- Addressing racial and ethnic disparities in maternal health requires evidence and policy solutions related to structural issues such systemic racism, which this project is poised to lead and succeed.

Weaknesses

- While the project leverages lived experiences of birthing individuals through deep community ties and has important policy implications, there seems to be a missed opportunity to include broader sociocultural and legal issues and corresponding scenarios (e.g., access to abortion care and systemic biases relevant to such care) in the research plan.

2. Investigator(s):

Strengths

- This project has the necessary intellectual, cultural, and regional community expertise needed to execute and translate the research findings.

Weaknesses

- None noted.

3. Innovation:

Strengths

- Using a mixed-methods approach, this project will examine multi-level systemic racism related to maternal health disparities and provide crucial evidence for relevant policy efforts.

Weaknesses

- None noted.

4. Approach:

Strengths

- State and hospital level indices of structural racism are appropriately defined in the context of the project objectives.
- The conceptual framework for the project is well-grounded and established in the field.
- The formulations and approach to study the impact of workforce diversity on the association between systemic racism and maternal outcomes are well-designed and appropriately applied.

Weaknesses

- None noted.

5. Environment:

Strengths

- The project has the necessary resources and connections to be successful.

Weaknesses

- None noted.

Study Timeline:

Strengths

- None noted.

Weaknesses

- None noted.

Protections for Human Subjects:

Acceptable Risks and Adequate Protections

Inclusion Plans:

- Sex/Gender: Distribution justified scientifically
- Race/Ethnicity: Distribution justified scientifically
- For NIH-Defined Phase III trials, Plans for valid design and analysis: Not applicable
- Inclusion/Exclusion Based on Age: Distribution justified scientifically

Vertebrate Animals:
Not Applicable (No Vertebrate Animals)

Biohazards:
Not Applicable (No Biohazards)

Resource Sharing Plans:
Acceptable

Authentication of Key Biological and/or Chemical Resources:
Not Applicable (No Relevant Resources)

Budget and Period of Support:
Recommend as Requested

THE FOLLOWING SECTIONS WERE PREPARED BY THE SCIENTIFIC REVIEW OFFICER TO SUMMARIZE THE OUTCOME OF DISCUSSIONS OF THE REVIEW COMMITTEE, OR REVIEWERS' WRITTEN CRITIQUES, ON THE FOLLOWING ISSUES:

PROTECTION OF HUMAN SUBJECTS: ACCEPTABLE

INCLUSION OF WOMEN PLAN: ACCEPTABLE

INCLUSION OF MINORITIES PLAN: ACCEPTABLE

INCLUSION ACROSS THE LIFESPAN: ACCEPTABLE

COMMITTEE BUDGET RECOMMENDATIONS: The budget was recommended as requested.

Footnotes for 1 U54 HD113172-01; PI Name: Reddy, Uma

NIH has modified its policy regarding the receipt of resubmissions (amended applications).See Guide Notice NOT-OD-18-197 at https://grants.nih.gov/grants/guide/notice-files/NOT-OD-18-197.html. The impact/priority score is calculated after discussion of an application by averaging the overall scores (1-9) given by all voting reviewers on the committee and multiplying by 10. The criterion scores are submitted prior to the meeting by the individual reviewers assigned to an application, and are not discussed specifically at the review meeting or calculated into the overall impact score. Some applications also receive a percentile ranking. For details on the review process, see http://grants.nih.gov/grants/peer_review_process.htm#scoring.

Principal Investigators (continued from first page):

RODRIGUEZ, EMILIE
SAFFORD, MONIKA M
ST. CLAIR, VICTORIA
TAYLOR, JACQUELYN Y
TAITT, SIMMONE

MEETING ROSTER

Center for Scientific Review Special Emphasis Panel
CENTER FOR SCIENTIFIC REVIEW
RFA-HD-23-035: Maternal Health Research Centers of Excellence

ZRG1 CTH-F (71)
04/11/2023 - 04/12/2023

Notice of NIH Policy to All Applicants: Meeting rosters are provided for information purposes only. Applicant investigators and institutional officials must not communicate directly with study section members about an application before or after the review. Failure to observe this policy will create a serious breach of integrity
in the peer review process, and may lead to actions outlined in NOT-OD-22-044 at https://grants.nih.gov/grants/guide/notice-files/NOT-OD-22-044.html, including removal of the application from immediate review.

CHAIRPERSON(S)

GRAY, KATHRYN JOHNSON, MD, PHD
PHYSICIAN-SCIENTIST
MATERNAL-FETAL MEDICINE
DEPARTMENT OF OBSTETRICS, GYNECOLOGY AND REPRODUCTIVE BIOLOGY
BRIGHAM AND WOMEN'S HOSPITAL
BOSTON, MA 02115

MEMBERS

AFABLE, AIMEE, PHD
ASSOCIATE DEAN AND ASSOCIATE PROFESSOR
DEPARTMENT OF COMMUNITY HEALTH SCIENCES
SCHOOL OF PUBLIC HEALTH
SUNY DOWNSTATE HEALTH SCIENCE UNIVERSITY
BROOKLYN, NY 11203

AHMADZIA, HOMA KHORRAMI, MD
ASSOCIATE PROFESSOR AND DIRECTOR OF MATERNAL-FETAL MEDICINE RESEARCH
DEPARTMENT OF OBSTETRICS AND GYNECOLOGY
SCHOOL OF MEDICINE AND HEALTH SCIENCES
THE GEORGE WASHINGTON UNIVERSITY
WASHINGTON, DC 20037

ANANTH, CANDE V., PHD
PROFESSOR AND VICE CHAIR FOR ACADEMIC AFFAIRS
DEPARTMENT OF OBSTETRICS, GYNECOLOGY, AND REPRODUCTIVE SCIENCES
ROBERT WOOD JOHNSON MEDICAL SCHOOL
RUTGERS BIOMEDICAL AND HEALTH SCIENCES
NEW BRUNSWICK, NJ 10032

ANDREWS, ARTHUR, PHD
ASSOCIATE PROFESSOR
DEPARTMENT OF PSYCHOLOGY
INSTITUTE FOR ETHNIC STUDIES
COLLEGE OF ARTS AND SCIENCES
UNIVERSITY OF NEBRASKA-LINCOLN
LINCOLN, NE 68588

BAILIT, JENNIFER L, MD
PROFESSOR
DEPARTMENT OF OBSTETRICS AND GYNECOLOGY
METROHEALTH MEDICAL CENTER
SCHOOL OF MEDICINE
CASE WESTERN RESERVE UNIVERSITY
CLEVELAND, OH 44106

BLEDSOE, SARAH ELIZABETH, PHD
ASSOCIATE PROFESSOR/CO-DIRECTOR
SCHOOL OF SOCIAL WORK
UNIVERSITY OF NORTH CAROLINA AT CHAPEL HILL
CHAPEL HILL, NC 27517

BORDERS, ANN E.B., MD
CLINICAL ASSOCIATE PROFESSOR
DEPARTMENT OF OBSTETRICS AND GYNECOLOGY
DIVISION OF MATERNAL FETAL MEDICINE
NORTHSHORE UNIVERSITY HEALTHSYSTEM
EVANSTON, IL 60201

BOULET, SHEREE L, DRPH
ASSOCIATE PROFESSOR
EMORY UNIVERSITY
SCHOOL OF MEDICINE
DEPARTMENT OF GYNECOLOGY AND OBSTETRICS
ATLANTA, GA 30303

BUDHWANI, HENNA, PHD
PROFESSOR
CENTER OF POPULATION SCIENCES FOR HEALTH EQUITY
COLLEGE OF NURSING
FLORIDA STATE UNIVERSITY
TALLAHASSEE, FL 32306

BYATT, NANCY, DO
PROFESSOR
DEPARTMENT OF PSYCHIATRY, OBSTETRICS AND GYNECOLOGY
UNIVERSITY OF MASSACHUSETTS MEDICAL SCHOOL
SHREWSBURY, MA 01545

CHARLTON, BRITTANY MICHELLE, DSC
ASSOCIATE PROFESSOR
DEPARTMENT OF POPULATION MEDICINE
HARVARD PILGRIM HEALTHCARE INSTITUTE
HARVARD MEDICAL SCHOOL
BOSTON, MA 02115

EDMONDS, JOYCE, PHD
ASSOCIATE PROFESSOR
CONNELL SCHOOL OF NURSING
BOSTON COLLEGE
CEHSTNUT HILL, MA 02467

EL AYADI, ALISON M, SCD
ASSOCIATE PROFESSOR
DEPARTMENTS OF OBSTETRICS, GYNECOLOGY, REPRODUCTIVE SCIENCES, EPIDEMIOLOGY, BIOSTATISTICS
BIXBY CENTER FOR GLOBAL REPRODUCTIVE HEALTH
UNIVERSITY OF CALIFORNIA, SAN FRANCISCO
SAN FRANCISCO, CA 94143

FARRELL, RUTH, MD
ASSOCIATE PROFESSOR
DEPARTMENT OF SUBSPECIALTY CARE FOR WOMEN'S HEALTH
CLEVELAND CLINIC
CLEVELAND, OH 44195

GREGORY, KIMBERLY D, MD
VICE CHAIR
DIVISION OF MATERNAL FETAL MEDICINE
DEPARTMENT OF OBSTETRICS AND GYNECOLOGY
CEDARS-SINAI MEDICAL CENTER
LOS ANGELES, CA 90048

HAAS, DAVID M., MD
PROFESSOR
DEPARTMENT OF OBSTETRICS & GYNECOLOGY
SCHOOL OF MEDICINE
INDIANA UNIVERSITY
INDIANAPOLIS, IN 46202

HEERMAN, WILLIAM, MD
ASSOCIATE PROFESSOR OF PEDIATRICS AND INTERNAL MEDICINE
CHIEF, SECTION OF INTERNAL MEDICINE AND PEDIATRICS
DIVISION OF GENERAL PEDIATRICS
VANDERBILT UNIVERSITY MEDICAL CENTER
NASHVILLE, TN 37232

HIMES, KATHERINE P, MD
ASSOCIATE PROFESSOR
DEPARTMENT OF OB/GYN AND REPRODUCTIVE SCIENCES
DIVISION OF MATERNAL-FETAL MEDICINE
MAGEE-WOMEN'S HOSPITAL OF UPMC
UNIVERSITY OF PITTSBURGH
PITTSBURGH, PA 15213

JOHNSON, SHARON D, PHD
DEAN AND PROFESSOR
SCHOOL OF SOCIAL WORK
UNIVERSITY OF MISSOURI, ST LOUIS
ST LOUIS, MO 63121-4499

JOSEPH, NATARIA TENNILLE, PHD
ASSOCIATE PROFESSOR
DEPARTMENT OF PSYCHOLOGY
SOCIAL SCIENCE DIVISION
PEPPERDINE UNIVERSITY
MALIBU, CA 90263

KELLEHER, SAMANTHA PARKER, PHD
ASSOCIATE PROFESSOR
DEPARTMENT OF EPIDEMIOLOGY
SCHOOL OF PUBLIC HEALTH
BOSTON UNIVERSITY
BOSTON, MA 02118

LINDSETH, GLENDA N, PHD
CHESTER FRITZ DISTINGUISHED PROFESSOR AND ASSOCIATE DEAN FOR RESEARCH
COLLEGE OF NURSING
UNIVERSITY OF NORTH DAKOTA
GRAND FORKS, ND 58201

LOGSDON, M CYNTHIA, PHD
PROFESSOR
SCHOOL OF NURSING
UNIVERSITY OF LOUISVILLE
LOUISVILLE, KY 40292

LORD, SARAH E, PHD
ASSOCIATE PROFESSOR
DISSEMINATION AND IMPLEMENTATION CORE
CENTER FOR TECHNOLOGY AND BEHAVIORAL HEALTH
DARTMOUTH CENTER FOR IMPLEMENTATION SCIENCE
GEISEL SCHOOL OF MEDICINE AT DARTMOUTH COLLEGE
GEISEL SCHOOL OF MEDICINE AT DARTMOUTH COLLEGE
LEBANON, NH 03766

LYNDON, AUDREY L, PHD
DEAN AND PROFESSOR
RORY MEYERS COLLEGE OF NURSING
NEW YORK UNIVERSITY
NEW YORK, NY 10010

MA, TONY XUYEN, MS
PRESIDENT
BENTEN TECHNOLOGIES, INC
MANASSAS, VA 20110

MAGIDSON, JESSICA F, PHD
ASSOCIATE PROFESSOR
DEPARTMENT OF PSYCHOLOGY
UNIVERSITY OF MARYLAND
COLLEGE PARK, MD 20742

MELLMAN, THOMAS A, MD
PROFESSOR
DEPARTMENT OF PSYCHIATRY AND BEHAVIORAL SCIENCES
COLLEGE OF MEDICINE
HOWARD UNIVERSITY
WASHINGTON, DC 20060

MISRA, DAWN P, PHD
PROFESSOR AND CHAIR
DEPARTMENT OF EPIDEMIOLOGY AND BIOSTATISTICS
COLLEGE OF HUMAN MEDICINE
MICHIGAN STATE UNIVERSITY
EAST LANSING, MI 48824

MUZIK, MARIA, MD
ASSOCIATE PROFESSOR
DEPARTMENT OF PSYCHIATRY
DEPARTMENT OF OBSTETRICS AND GYNECOLOGY
UNIVERSITY OF MICHIGAN
ANN ARBOR, MI 48109

OATES, GABRIELA R, PHD
ASSOCIATE PROFESSOR
DEPARTMENT OF PEDIATRICS
HEERSINK SCHOOL OF MEDICINE
THE UNIVERSITY OF ALABAMA AT BIRMINGHAM
BIRMINGHAM, AL 35233

PHELAN, SUZANNE, PHD
PROFESSOR AND DIRECTOR
DEPARTMENT OF KINESIOLOGY AND PUBLIC HEALTH
CALIFORNIA POLYTECHNIC STATE UNIVERSITY
SAN LUIS OBISPO, CA 93407

SANTOS, HUDSON, PHD
PROFESSOR AND ASSOCIATE DEAN FOR RESEARCH
SCHOOL OF NURSING & HEALTH STUDIES
UNIVERSITY OF MIAMI
CORAL GABLES, FL 33146

SHALOWITZ, MADELEINE ULLMAN, MD
PROFESSOR
DEPARTMENT OF PEDIATRICS
RUSH UNIVERSITY MEDICAL CENTER
CHICAGO, IL 60612

SHEPARD, DONALD SLOANE, PHD
PROFESSOR
SCHNEIDER INSTITUTES FOR HEALTH POLICY
HELLER SCHOOL FOR SOCIAL POLICY AND MANAGEMENT
BRANDEIS UNIVERSITY
WALTHAM, MA 02454

SMITH, SHARLA ANNETTE, PHD
ASSOCIATE PROFESSOR
DEPARTMENT OF PREVENTIVE MEDICINE & PUBLIC HEALTH
SCHOOL OF MEDICINE
THE UNIVERSITY OF KANSAS, WICHITA
WICHITA, KS 67214

SOCKOLOW, PAULINA S, DRPH
ASSOCIATE PROFESSOR
HEALTH SYSTEMS AND SCIENCES RESEARCH
COLLEGE OF NURSING AND HEALTH PROFESSIONS
DREXEL UNIVERSITY
PHILADELPHIA, PA 19102

STROBINO, DONNA M., PHD
PROFESSOR AND VICE CHAIR OF EDUCATION
POPULATION, FAMILY AND REPRODUCTIVE HEALTH
SCHOOL OF HYGIENE AND PUBLIC HEALTH
JOHNS HOPKINS UNIVERSITY
BALTIMORE, MD 21205

SUBBIAN, VIGNESH, PHD
ASSOCIATE PROFESSOR
DEPARTMENT OF BIOMEDICAL ENGINEERING
COLLEGE OF ENGINEERING
UNIVERSITY OF ARIZONA
TUSCAN, AZ 85721

TAYLOR, BRANDIE DEPAOLI, PHD
ASSOCIATE PROFESSOR AND DIRECTOR
DEPARTMENT OF OBSTETRICS & GYNECOLOGY
SCHOOL OF MEDICINE
UNIVERSITY OF TEXAS MEDICAL BRANCH
GALVESTON, TX 77555-1068

WASHIO, YUKIKO, PHD
RESEARCHER
RTI INTERNATIONAL
MEDIA, PA 19063

WILSON, PATRICK ALAN-DAVID, PHD
PROFESSOR
DEPARTMENT OF PSYCHOLOGY
UNIVERSITY OF CALIFORNIA, LOS ANGELES
LOS ANGELES, CA 90095

WINTERSTEIN, ALMUT G, PHD
PROFESSOR
COLLEGE OF PHARMACY
UNIVERISTY OF FLORIDA
GAINESVILLE, FL 32608

YAO, XIAOXI, PHD
ASSOCIATE PROFESSOR
DEPARTMENT OF HEALTH SCIENCES RESEARCH
MAYO CLINIC
ROCHESTER, MN 55905

MAIL REVIEWER(S)

MESSER, LYNNE C, PHD
ASSOCIATE PROFESSOR
SCHOOL OF COMMUNITY HEALTH
COLLEGE OF URBAN AND PUBLIC AFFAIRS
PORTLAND STATE UNIVERSITY
PORTLAND, OR 97207

SCIENTIFIC REVIEW OFFICER

BELLINGER, JESSICA, PHD
SCIENTIFIC REVIEW ADMINISTRATOR
CENTER FOR SCIENTIFIC OF REVIEW
NATIONAL INSTITUTES OF HEALTH
BETHESDA, MD 20892

EXTRAMURAL SUPPORT ASSISTANT

PANIAGUA, MIGUEL ALEJANDRO
LEAD EXTRAMURAL ASSISTANT
CENTER FOR SCIENTIFIC REVIEW
NATIONAL INSTITUES OF HEALTH
BETHESDA, MD 20892

Consultants are required to absent themselves from the room during the review of any application if their presence would constitute or appear to constitute a conflict of interest.
